# Vitamin D_3_ Attenuates Neuropathic Pain via Suppression of Mitochondria‐Associated Ferroptosis by Inhibiting PKCα/NOX4 Signaling Pathway

**DOI:** 10.1111/cns.70067

**Published:** 2024-09-27

**Authors:** Wencui Zhang, Shangchen Yu, Bo Jiao, Caixia Zhang, Kaiwen Zhang, Baowen Liu, Xianwei Zhang

**Affiliations:** ^1^ Department of Anesthesiology and Pain Medicine, Hubei Key Laboratory of Geriatric Anesthesia and Perioperative Brain Health, and Wuhan Clinical Research Center for Geriatric Anesthesia Tongji Hospital, Tongji Medical College, Huazhong University of Science and Technology Wuhan China

**Keywords:** ferroptosis, GABAergic interneurons, mitochondrial dysfunction, neuropathic pain, PKCα/NOX4 signaling, vitamin D_3_

## Abstract

**Aims:**

Neuropathic pain remains a significant unmet medical challenge due to its elusive mechanisms. Recent clinical observations suggest that vitamin D (VitD) holds promise in pain relief, yet its precise mechanism of action is still unclear. This study explores the therapeutical role and potential mechanism of VitD_3_ in spared nerve injury (SNI)‐induced neuropathic pain rat model.

**Methods:**

The analgesic effects and underlying mechanisms of VitD_3_ were evaluated in SNI and naïve rat models. Mechanical allodynia was assessed using the Von Frey test. Western blotting, immunofluorescence, biochemical assay, and transmission electron microscope (TEM) were employed to investigate the molecular and cellular effects of VitD_3_.

**Results:**

Ferroptosis was observed in the spinal cord following SNI. Intrathecal administration of VitD_3_, the active form of VitD, activated the vitamin D receptor (VDR), suppressed ferroptosis, and alleviated mechanical nociceptive behaviors. VitD_3_ treatment preserved spinal GABAergic interneurons, and its neuroprotective effects were eliminated by the ferroptosis inducer RSL3. Additionally, VitD_3_ mitigated aberrant mitochondrial morphology and oxidative metabolism in the spinal cord. Mechanistically, VitD_3_ inhibited SNI‐induced activation of spinal PKCα/NOX4 signaling. Inhibition of PKCα/NOX4 signaling alleviated mechanical pain hypersensitivity, accompanied by reduced ferroptosis and mitochondrial dysfunction in SNI rats. Conversely, activation of PKCα/NOX4 signaling in naïve rats induced hyperalgesia, ferroptosis, loss of GABAergic interneurons, and mitochondrial dysfunction in the spinal cord, all of which were reversed by VitD_3_ treatment.

**Conclusions:**

Our findings provide evidence that VitD_3_ attenuates neuropathic pain by preserving spinal GABAergic interneurons through the suppression of mitochondria‐associated ferroptosis mediated by PKCα/NOX4 signaling, probably via VDR activation. VitD, alone or in combination with existing analgesics, presents an innovative therapeutic avenue for neuropathic pain.

## Graphical abstract

Schematic diagram of the analgesic mechanism of vitamin D_3_ in neuropathic pain. Vitamin D_3_ protects GABAergic interneurons against ferroptosis through activation of VDR and repression of PKCα‐NOX4 signaling‐mediated mitochondrial dysfunction.

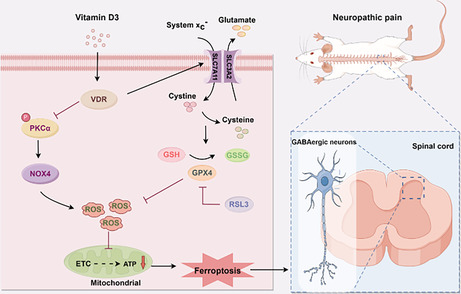

**Abbreviations**
ATP5F1A: ATP synthase F1 subunit alpha
ETC: electron transport chain
GABA: gama‐aminobutyric acid
GAD65: glutamic acid decarboxylase 65
GFAP: glial fibrillary acidic protein
GPX4: glutathione‐dependent peroxidase
GSH: glutathione
IBA1: ionized calcium‐binding adapter molecule 1
MTCO1: mitochondrially encoded cytochrome c oxidase I
NDUFB8: NADH: Ubiquinone Oxidoreductase Subunit B8
NeuN: neuronal nuclei
NOX4: NADPH oxidase 4
PKCα: protein kinase C alpha
PWT: paw withdrawal threshold
ROS: reactive oxygen species
SDHB: succinate dehydrogenase complex iron sulfur subunit B
SLC7A11: subunit solute carrier family 7 member 11
SNI: spared nerve injury
UQCRC2: ubiquinol‐cytochrome c reductase core protein 2
VDR: vitamin D receptor
VitD_3_
: vitamin D_3_

## 1 Introduction

Neuropathic pain is a debilitating pathological pain condition resulting from lesions or disease of the somatosensory nervous system [1]. It represents a major public health burden, affecting 7%–10% of the general population and imposing a considerable negative impact on patients' somatic function and quality of life [2]. Due to the limited understanding of the intricate mechanism underlying neuropathic pain, current pharmacological therapies are woefully inadequate in relieving pain syndromes of suffers and often accompanied by significant adverse effects [3]. Consequently, there is an urgent need to elucidate the pathophysiology of neuropathic pain, enabling the development of mechanism‐based therapeutics that maximize analgesic efficacy while minimizing side effects.

Compelling evidence have proposed an imbalance between excitatory and inhibitory signals within spinal circuits in neuropathic pain [4]. Specifically, reduction in the inhibitory neurotransmitter gama‐aminobutyric acid (GABA) and the loss of GABAergic interneurons in the spinal dorsal horn are associated with hyperalgesia and allodynia [5]. Ding et al. recently demonstrated that inhibiting ferroptosis‐like cell death can prevent the loss of spinal GABAergic interneurons and alleviate bone cancer pain [6]. Ferroptosis, a new type of regulated cell death driven by iron‐dependent lipid peroxidation, has been implicated in various pathological conditions, including neurodegenerative disease, ischemic/reperfusion injury (IR), stroke, and cancer [7, 8]. Ferroptosis is characterized by oxidative stress resulting from reactive oxygen species (ROS) accumulation, which inhibits the activity of the cystine‐glutamate antiporter (system Xc‐), leading to glutathione (GSH) depletion and inactivation of glutathione‐dependent glutathione peroxidase (GPX4), thus disrupting cellular redox homeostasis [9]. Accumulating evidence indicates the pivotal role of ferroptosis in the development of neuropathic pain, and highlighting that ferroptosis is a prospective therapeutic target [10, 11, 12].

Mitochondria are responsible for energy supply through oxidative phosphorylation via a series of electron transport chain (ETC) complexes. It is also known as the principal loci for ROS production, playing a critical role in regulating oxidative stress and cell death [13]. Ferroptosis is closely associated with irreversibly damage to the mitochondrial morphology and function, which in turn triggered ferroptosis [14]. Despite the observed destroyed mitochondrial morphology, the functional relevance of mitochondrial damage in ferroptosis under neuropathic pain states has not been investigated yet.

NADPH oxidases (nicotinamide adenine dinucleotide phosphate oxidase, NOXs), a family enzymes that generate ROS [15]. NOXs consist of seven members: NOX1–5 and the dual oxidases Duox1 and Duox2. Specifically, NOX4 is constitutively active and has been identified in nociceptive primary afferent neurons [16]. NOX4‐derived ROS can impair mitochondrial metabolism by inhibiting mitochondrial respiration and ATP production, leading to ferroptosis of astrocytes [17]. Protein kinase C α (PKCα) is an important regulator of ion channels coupled with numerous signaling cascades, and interferes with multiple processes, such as apoptosis, autophagy, cellular differentiation, and metabolism [18]. PKCα serve as an upstream activator of NOX4 through phosphorylation, and PKCα/NOX4 signaling pathway has been shown to regulate ferroptotic signaling cascade [19, 20]. Yet, the role of PKCα/NOX4 signaling in mitochondrial function and ferroptosis in the progression of neuropathic pain has not been explored.

Vitamin D, a fat‐soluble vitamin, is essential for calcium homeostasis and bone metabolism. The bioactive form of vitamin D is vitamin D_3_ (VitD_3_, also known as calcitriol), which exhibit antioxidant and neuroprotective properties through binding with the vitamin D receptor (VDR), potentially useful against neurodegenerative disease [21, 22]. An increasing number of observational studies have linked vitamin D deficiency with multiple painful manifestations, such as diabetic neuropathy, postherpetic neuralgia (PHN), bone pain, and fibromyalgia [23, 24, 25]. Vitamin D supplementation has demonstrated clinical benefits in patients with fibromyalgia or neuropathic pain [25, 26, 27]. However, the mechanism by which vitamin D modulates pain is not well understood. In this study, we utilized spared nerve injury (SNI)‐induced rat model to investigate the potential of VitD_3_ in treating neuropathic pain and to elucidate the possible molecular mechanisms.

## 2 Materials and Methods

### 2.1 Animals

Adult male Sprague–Dawley rats (weighing 200–240 g, supplied from Tongji Hospital, Tongji Medical College, Huazhong University of Science and Technology, Wuhan, China) were used in this study. Rats were randomly grouped and housed in an environment with a temperature (23 ± 2°C), relative humidity (45%–65%), lighting (12‐h light/dark cycle). All rats must acclimate to standard conditions for at least 7 days upon arrival, with free access to food and water. Animal procedures were approved by the Ethics Committee of Tongji hospital, Tongji Medical College, Huazhong University of Science and Technology (Number: TJH‐202306019) and in accordance with the National Institutes of Health Guidelines for the Care and Use of Laboratory Animals and ARRIVE Guidelines for Reporting Animal Research. All experiments were completed in the Experimental Medicine Research Center of Tongji hospital.

### 2.2 Chemicals and Reagents

VitD_3_ (Calcitriol, cat: HY‐10002, purity > 95%) was dissolved in a solution containing 10% DMSO and 90% corn oil. RSL3 (cat: HY‐100218A), an activator of ferroptosis through inactivate GPX4, was dissolved in 10% DMSO and 90% corn oil. The PKCα inhibitor bisindolylmaleimide I (BIM‐1, cat: HY‐13867) was dissolved in 10% DMSO and 40% PEG300 and 5% Tween‐80 in sterile saline. The PKCα agonist phorbol 12‐myristate 13‐acetate (PMA, cat: HY‐18739) was dissolved in 10% DMSO and 40% PEG300 and 5% Tween‐80 in sterile saline. The vehicles were formulated on the basis of the corresponding solvent medium. All the chemicals employed in the present study were purchased from Med Chem Express (Shanghai, China). Iron Assay Kit (cat: A039‐2‐1), malondialdehyde (MDA) Assay Kit (cat: A003‐1‐1), ROS Assay Kit (cat: E004‐1‐1), Superoxide Dismutase (SOD) Assay Kit (cat: A001‐1‐1), GSH Assay Kit (cat: A006‐2‐1), and ATP Assay Kit (cat: A095‐1‐1) were purchased from Jiancheng Biology (Nanjing, China). Perl's staining Kit was purchased from Servicebio (Wuhan, China). The antibodies used in the molecular studies were showed in Table 1.

**TABLE 1:** Primary and secondary antibodies for western blot and immunofluorescence.

Antibody	Provider	Host	Catalog number	Dilution for WB	Dilution for IF
VDR	Proteintech	Mouse	67,192‐1‐Ig	1:2000	
	Abclonal	Rabbit	A2194		1:50
p‐PKCα	Affinity	Rabbit	AF8396	1:1000	1:50
PKCα	Abclonal	Rabbit	A11107	1:1000	
NOX4	Proteintech	Rabbit	14,347‐1‐AP	1:2000	1:50
SLC7A11	Abclonal	Rabbit	A2413	1:2000	
GPX4	Abclonal	Rabbit	A11243	1:2000	
GAD65	Zen‐bio	Mouse	201,308	1:1000	
NDUFB8	Abclonal	Rabbit	A19732	1:1000	
SDHB	Abclonal	Rabbit	A1809	1:1000	
UQCRC2	Abclonal	Rabbit	A4366	1:2000	
MTCO1	Abclonal	Rabbit	A17889	1:1000	
ATP5A1	Abclonal	Rabbit	A11217	1:2000	
β‐Actin	Abclonal	Rabbit	AC038	1:10000	
IBA‐1	Gene Tex	Mouse	GTX632426		1:200
NeuN	Abcam	Mouse	Ab104224		1:200
GFAP	CST	Mouse	#3670		1:200
Anti‐rabbit IgG HRP	Abclonal	Goat	AS014	1:5000	
Anti‐mouse IgG HRP	Abclonal	Goat	AS003	1:5000	
Coralite488‐conjugated anti‐mouse IgG	Proteintech	Goat	SA00013‐5		1:200
Coralite594‐conjugated anti‐rabbit IgG	Proteintech	Mouse	SA00013‐8		1:200

Abbreviations: ATP5A1 (also term as ATP5F1A), ATP synthase F1 subunit alpha; GAD65, glutamic acid decarboxylase 65; GFAP, glial fibrillary acidic protein; GPX4, glutathione peroxidase 4; HRP, horseradish peroxidase; IBA1, ionized calcium‐binding adapter molecule 1; MTCO1, mitochondrially encoded cytochrome c oxidase I; NDUFB8, NADH: Ubiquinone Oxidoreductase Subunit B8; NeuN, neuronal nuclei; NOX4, NADPH oxidase 4; PKCα, protein kinase C alpha; SDHB, succinate dehydrogenase complex iron sulfur subunit B; SLC7A11, subunit solute carrier family 7 member 11; UQCRC2, ubiquinol‐cytochrome c reductase core protein 2; VDR, vitamin D receptor.

### 2.3 Experimental Design

In the present study, all rats were randomly allocated to different groups using a computer‐generated random number table. All the experimental procedures and analyses were blindly performed by researchers unaware of the group assignments. Five main experiments were designed to investigate the analgesic mechanism of VitD_3_ in neuropathic pain.

#### 2.3.1 Experiment 1

Rats were randomly assigned to the sham or SNI group. Paw withdrawal thresholds (PWTs) were measured on Days 3, 7, and 14 after surgery. Then, the L4–L6 spinal cords from the sham group and the SNI group at 3, 7, and 14 days after surgery were collected for western blot and Kit detection. We chose rats 7 days after SNI for double immunofluorescence colocalization and Perl's staining.

#### 2.3.2 Experiment 2

To verify whether a single dose of calcitriol could attenuate mechanical allodynia, rats were randomly divided into four groups, respectively: SNI + Vehicle; SNI + 0.5 μg calcitriol; SNI + 1 μg calcitriol; SNI + 2 μg calcitriol. Vehicle and different doses of calcitriol were intrathecally (i.t.) administrated on 7 days after surgery. To assess the effect of continuous calcitriol injection on mechanical allodynia in SNI, rats were randomly divided into four groups: Sham + Vehicle; Sham + calcitriol; SNI + Vehicle; SNI + calcitriol. Calcitriol (1 μg, i.t.) was administrated once daily for 5 consecutive days, starting on the 7th day after surgery.

#### 2.3.3 Experiment 3

To investigate whether the ferroptosis agonist RSL3 could blocked the protective effect of calcitriol, rats were randomly divided into 2 groups:SNI + calcitriol and SNI + calcitriol + RSL3. Calcitriol alone or co‐administrated with RSL3 (5 μg) was intrathecally injected once daily for 5 consecutive days, starting on the 7th after the surgery. The dose of RSL3 was chosen on the basis of unpublished data from our laboratory.

#### 2.3.4 Experiment 4

To investigate whether pharmacological inhibition of PKCα/NOX4 signaling pathway could attenuate mechanical allodynia, rats were randomly divided into four groups: Sham + Vehicle; Sham + BIM‐1; SNI + Vehicle; SNI + BIM‐1. BIM‐1 (100 μM) was intrathecally administrated into corresponding rats according to a previous study [28], from 7 to 11 days after surgery.

#### 2.3.5 Experiment 5

To study the role of PKCα/NOX4 signaling pathway in the analgesic effect of calcitriol, naïve rats were randomly divided into three groups: Vehicle, Vehicle + PMA, Vehicle + PMA + Calcitriol. A single dose of PMA (2 μg) or a combination of calcitriol were intrathecally injected into naïve rats. The dose of PMA was chosen on the basis of previous studies [29].

### 2.4 Neuropathic Pain Model

The SNI rat model of neuropathic pain was induced as previously described [30]. Briefly, the rats were anesthetized with 2.5% isoflurane and were placed in a left decubitus position; an incision of approximately 1 cm was made at the mid‐thigh level, the left sciatic nerve and its three branches (the common peroneal, tibial, sural nerves) were carefully exposed and separated. Then, the common peroneal and tibial nerves were ligated together and transected distally, leaving the sural nerve intact. The skin incision was sutured with 3.0 silk and disinfected. The sham‐operated animals underwent exactly the same procedure without nerve injury.

### 2.5 Behavioral Testing

The mechanical allodynia was evaluated by measuring the hind PWT in response to von Frey filament stimuli, as previously described [31]. In brief, rats were individually positioned in test cages with a mesh floor and habituated for 30 min before von Frey testing. The von Frey filaments ranging from 1.4 to 15 g were vertically applied in ascending order on the mid‐plantar of the hind paw, bending the filament for 3–5 s. A positive response was defined as quick paw withdrawal, licking, or shaking. The lowest force required to elicit a positive response was recorded as PWTs. All behavioral tests were performed between 9:00 a.m. and 6:00 p.m. by an investigator blinded to the experimental design.

### 2.6 Intrathecal Catheterization and Drug Administration

For intrathecal drug administration, the intrathecal catheterization was completed at least 5 days prior to sham or SNI surgery as previously reported [32]. Rats were deeply anesthetized with isoflurane (2.5%) and implanted with PE‐10 catheters (inner diameter 0.3 mm, outer diameter 0.6 mm) into the intrathecal space at the L4‐L6 level. The correct position of the catheter was confirmed by the tail‐flick response immediately following the insertion of the catheter. After surgery, 2% lidocaine (10 μL) was delivered through the intrathecal catheter, and both hind limbs of the rats showed temporary paralysis, indicating the success of catheterization. Animals with visible motor dysfunction were excluded from the experiment. In this study, we aimed to investigate a purely spinal mechanism. All drugs were intrathecally injected at 10 μL, followed by 10 μL saline for flushing. Detailed experimental plans for animal assignment and medication are outlined in the respective timeline diagram.

### 2.7 Western Blot

Under deep anesthetized, the L4‐L6 spinal cord was quickly removed and homogenized in ice‐cold RIPA lysis‐buffer containing a cocktail of protease and phosphatase inhibitors. The lysates were then centrifuged at 12,000 rpm at 4°C for 15 min, and the protein concentration of supernatants was measured using BCA method. Equal amounts of protein samples (40 μg) were separated by 10% sodium dodecyl sulfate–polyacrylamide gels electrophoresis (SDS‐PAGE) and transferred to polyvinylidene difluoride (PVDF) membranes (Millipore, Billerica, MA, USA). After blocking with 5% non‐fat milk for 2 h at RT, the membranes were incubated with the primary antibodies (Table 1) overnight at 4°C. Following washes in TBST three times, the blots were incubated with an HRP‐conjugated secondary antibody for 2 h at RT and visualized with Super Lumia ECL Plus HRP Substrate Kit (Abbkine). The bolts were scanned and quantified using the ChemiDoc XRS System (Bio‐Rad, CA, USA). The intensity of bands was normalized to the β‐Actin and expressed as fold changes over the control group. For successive antibody incubations, the primary and secondary antibodies were removed using a commercial stripping solution (Epizyme Biotech, Shanghai, China), and the membranes were re‐immunoblotted for additional molecules.

### 2.8 Paraffin Section Preparation

After deep anesthesia with 2.5% isoflurane, rats were intracardially perfused with ice‐cold 0.1 M PBS followed by 4% paraformaldehyde in PBS. Subsequently, the L4‐L6 spinal cord segments were harvested, fixed in 4% PFA at room temperature, dehydrated, and embedded in paraffin. Four‐micrometer thick paraffin sections were used for immunofluorescence and Perl's staining.

### 2.9 Double‐Labeling Immunofluorescence

After deparaffinization and hydration, the sections were microwaved in 0.01 M sodium citrate (PH 6.0) for antigen retrieval. The sections were penetrated with 0.3% TritonX‐100 for 15 min and blocked with 5% donkey serum for 1 h at RT. Then, the sections were incubated with the primary antibodies (Table 1) overnight at 4°C. After washing with PBS three times, the sections were incubated with the secondary antibodies (Table 1) for 2 h at RT. The nuclei were stained with DAPI for 5 min, and images were captured using a fluorescence microscope (BX51, OLYMPUS, Japan).

### 2.10 Perl's Staining and Iron Content Detection

Iron deposition was measured in spinal cord by Perl's staining. Perl's staining working solution was added to the deparaffinized and hydrated sections. Then, hemosiderin or ferric iron staining was observed under the microscope. The concentration of Fe^2+^ in the spinal cord expressed as nmol/g protein was measured with a kit assay according to the manufacturer's protocols.

### 2.11 ROS, MDA, GSH, and SOD Measurements

The contents of ROS, MAD, GSH, and SOD in spinal cord tissues were detected using commercial assay kits according to the manufacturer's protocol. The content of MDA, GSH, and SOD was expressed as nmol/g protein, μmol/g protein, and U/g protein, respectively.

### 2.12 Measurement of ATP Levels

The ATP levels of the spinal cord were calculated with an assay kit following the manufacturer's operation manual. The concentration of ATP was expressed in the form of μmol/g protein.

### 2.13 Transmission Electron Microscope

Rats were deeply anesthetized and transcardially perfused with 0.1 M PBS followed by 4% ice‐cold PFA, the spinal cord was dissected out and sliced into 1 mm^2^ sections. The sections were fixed in 2% glutaraldehyde, post‐fixed in 1% aqueous osmium tetraoxide for 2 h at RT, dehydrated in gradual ethanol, and embedded in epoxy resin. Ultrathin slides were obtained using ultra‐micro‐tome (Leica, Germany) and double‐stained with uranyl acetate and lead citrate. Images were captured by transmission electron microscopy (Thermo, USA).

### 2.14 Statistical Analysis

All experimental data are presented as mean ± SD and analyzed using GraphPad Prism version 6 (Graph Pad Software, San Diego, CA, USA). All data underwent normality testing using either the Shapiro–Wilk test. Differences in behavioral scores among groups were analyzed by two‐way repeated measures analysis of variance (ANOVA), followed by Bonferroni's post hoc test. In other trails, Student's *t*‐tests were used for comparisons between two groups, and one‐way ANOVA followed by the Bonferroni test was used for comparisons between multiple groups. For all statistical analyses, *p* < 0.05 was interpreted as statistically significant.

## 3 Results

### 3.1 SNI Induces Mechanical Allodynia and Ferroptosis in the Spinal Cord

**FIGURE 1:**
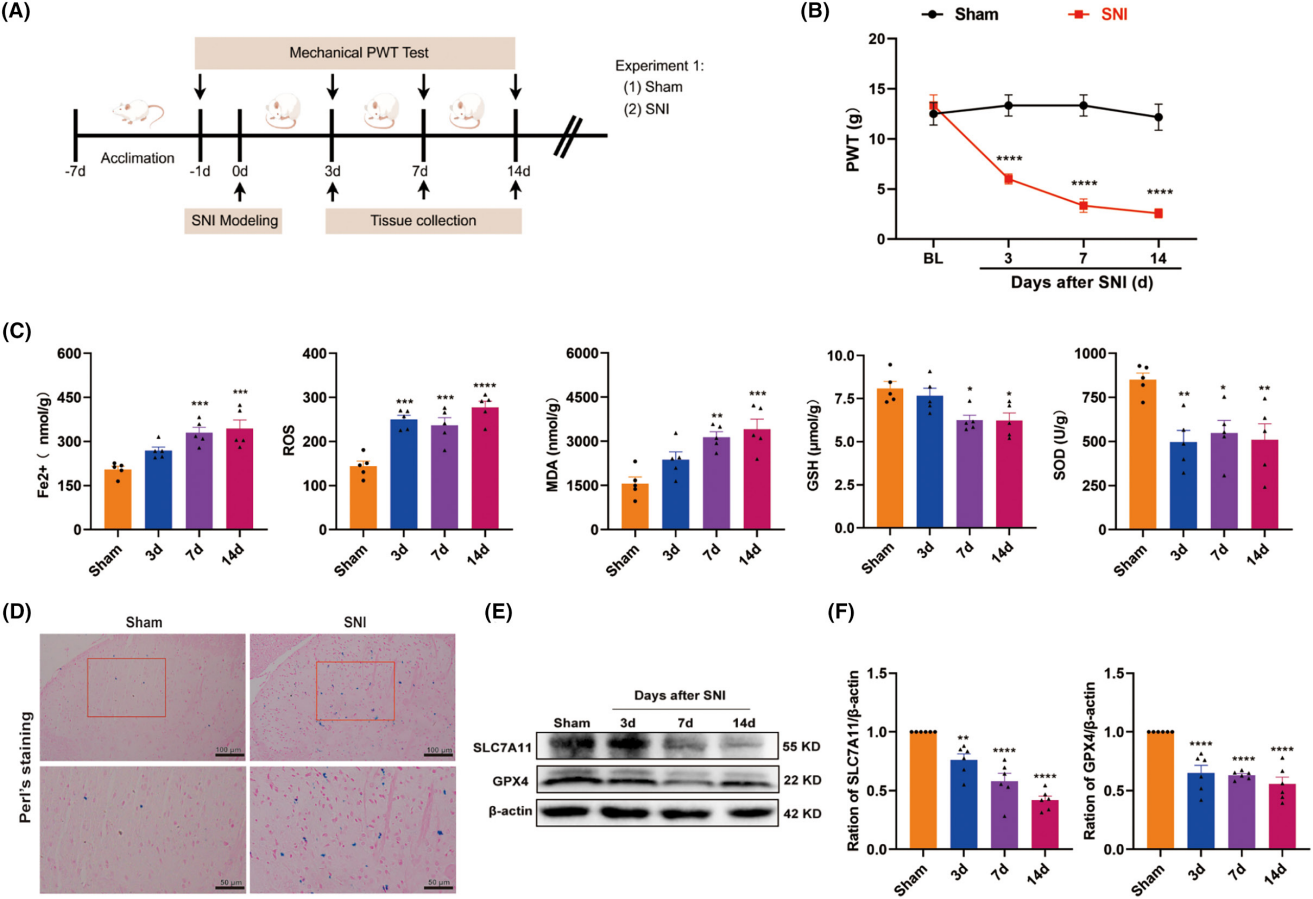
SNI‐induced mechanical allodynia and ferroptosis in the spinal cord. (A) The schematic timeline of the experimental procedure. (B) Mechanical allodynia evaluated by PWT at baseline and 3, 7, and 14 days after sham or SNI (*n* = 6 per group). ****p < 0.0001 versus sham group. (C) Time course of Fe^2+^, ROS, MDA, GSH, and SOD levels in the spinal cord of SNI rats (*n* = 5 per group). *p < 0.05, **p < 0.01, ***p < 0.001, ****p < 0.0001 versus sham group. (D) Representative pictures of Perl's staining in spinal cord were shown (*n* = 4 per group). (E, F) Time course of ferroptosis‐related proteins SLC7A11 and GPX4 in the spinal cord after SNI (*n* = 6 per group). β‐Actin was used as an internal control, **p < 0.01, ****p < 0.0001 versus sham group.

In this study, we adopt SNI rats as an animal model to investigate neuropathic pain, with a focus on mechanical allodynia. The timeline of the surgery and behavior tests is depicted in Figure 1A. There is no significant difference at baseline between the sham and SNI groups regarding the ipsilateral PWT. In contrast to the sham control, SNI rats exhibited a significant reduction in PWT from Day 3 to Day 14 after SNI (Figure 1B), indicating that the SNI surgery successfully evoked significant mechanical hypersensitivity.

To determine the potential involvement of ferroptosis in the development of SNI‐induced neuropathic pain, we first examined the time‐course expression of the biochemical hallmarks of ferroptosis in the spinal cord on Days 3, 7, and 14 after SNI. As shown in Figure 1C, the spinal levels of Fe^2+^, ROS, and MDA increased on Day 3 after SNI and continued to rise over time. Conversely, the activity of GSH and SOD, the main component of the antioxidant defense system in ferroptosis [33], persistently decreased in the spinal cord of SNI rats compared with the sham control. Perl's staining revealed significant iron deposition that was represented as blue spots in the ipsilateral spinal dorsal horn of SNI rats, when compared to the sham control (Figure 1D). We also observed the dynamic changes of key regulator of ferroptosis, such as SLC7A11 and GPX4, both of which significantly decreased in the spinal cord of SNI rats (Figure 1E,F). These results consistently suggest that ferroptosis is involved in the development of neuropathic pain at the spinal cord level.

### 3.2 The Analgesic Effect of VitD_3_ in SNI Rats

**FIGURE 2:**
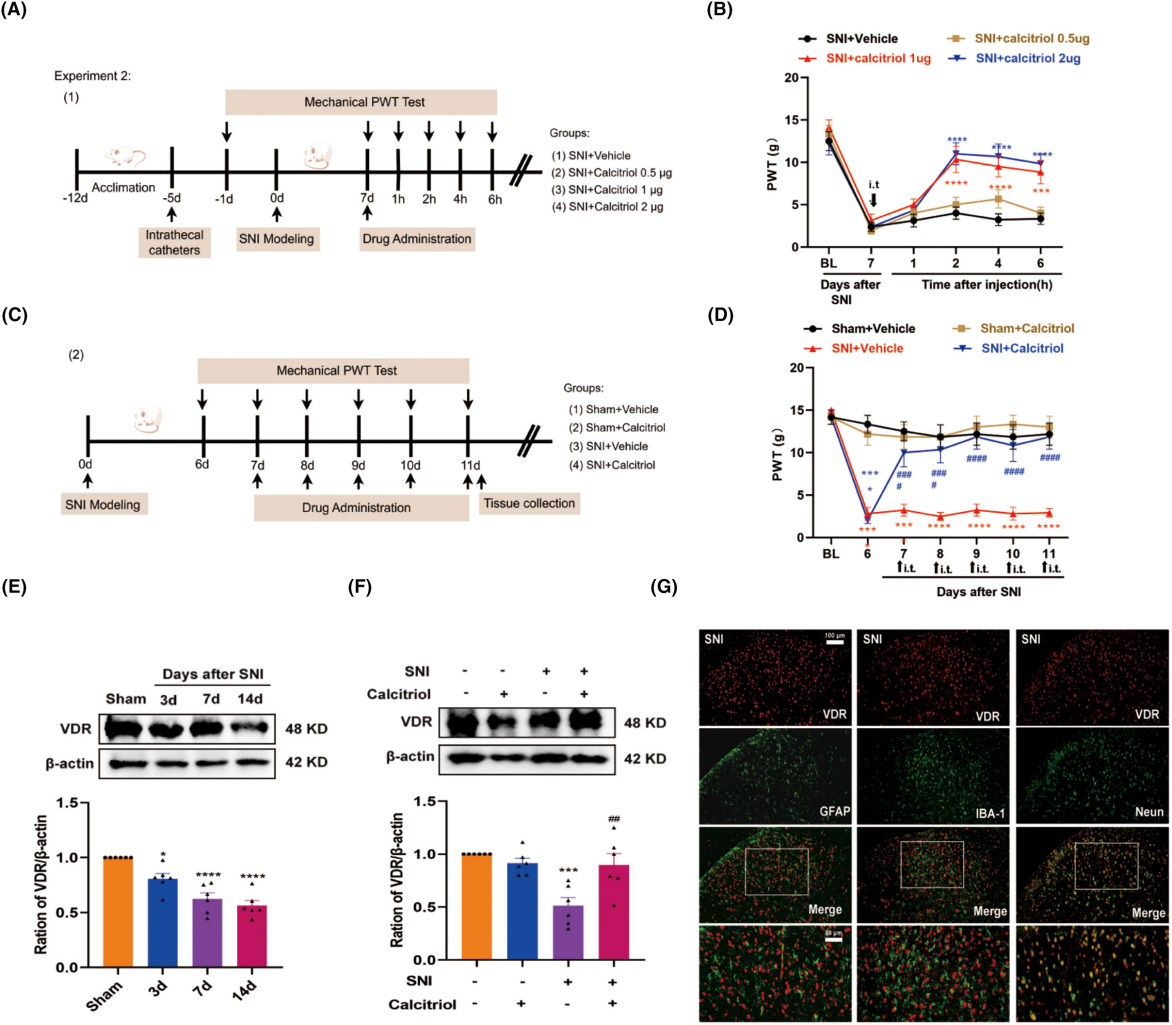
Analgesic effect of VitD_3_ on mechanical allodynia in SNI rats. (A, B) A single dose of calcitriol (0.5, 1 and 2 μg, i.t.) or vehicle was administrated on Day 7 following SNI. Mechanical allodynia was measured before injection and 1, 2, 4, and 6 h after injection (*n* = 6 per group). ***p < 0.001, ****p < 0.0001 versus SNI + Vehicle group. (C, D) Calcitriol (1 μg, i.t.) was given once daily from Day 7 to Day 11 following SNI. Mechanical allodynia was conducted at 2 h after calcitriol injection from Day 7 to Day 11 following SNI (*n* = 6 per group). ***p < 0.001, ****p < 0.0001 versus Sham + Vehicle group, ^####^ *p* < 0.0001 versus SNI + Vehicle group. (E) Time course of VDR in the spinal cord after SNI (*n* = 6 per group). *p < 0.05, ****p < 0.0001 versus sham group. (F) Representative blots and quantification of VDR in the spinal cord of different groups were presented (*n* = 6 per group). ***p < 0.001 versus Sham + Vehicle group, ^##^ *p* < 0.01 versus SNI + Vehicle group. (G) Double immunofluorescence of VDR (red) and GFAP (green), IBA1 (green), and NeuN (green) in the spinal cord of SNI rats (*n* = 4 per group). The white boxes indicated typical co‐staining cells. Scale bar: 100 and 50 μm.

To explore whether VitD_3_ could alleviate mechanical allodynia induced by SNI, calcitriol was intrathecally injected into sham and SNI rats. The timeline of this experiment is shown in Figure 2A. Firstly, a different selected dose of calcitriol (0.5, 1, or 2 μg) was given on Day 7 post‐surgery. The von Fery test was performed before calcitriol injection and at 1, 2, 4, and 6 h after the first injection. As illustrated in Figure 2B, calcitriol treatment significantly increased the ipsilateral PWT in SNI rats at an effective dose of 1 or 2 μg compared with the SNI + Vehicle group. The effect peaked at 2 h after administration and persisted for at least 4 h. However, a low dose of calcitriol (0.5 μg) was devoid of any activity. Then, to determine whether repeated treatment with calcitriol has cumulative analgesic effects, calcitriol (1 μg) was administrated once daily for 5 consecutive days starting from Day 7 (Figure 2C). The behavioral test was conducted before and 2 h after calcitriol injection. As shown in Figure 2D, repeated calcitriol injection significantly reversed the established mechanical allodynia in SNI rats without tolerance. These results indicate that VitD_3_ effectively ameliorates neuropathic pain in a dose‐dependent manner.

VitD_3_ exerts its biological functions through ligand activation of VDR, which is widely expressed in the peripheral and central neurons involved in pain‐sensing and processing [34]. Therefore, we examined the effect of calcitriol on VDR expression in the spinal cord of SNI rats. As shown in Figure 2E,F, western blot results displayed decreased levels of VDR caused by SNI, partially reversed by repeated treatment with calcitriol. Then, to identify the cell type expressing VDR in the spinal dorsal horn, we examined the colocalization of VDR with the markers specific for astrocytes (GFAP), microglial (IBA‐1), and neurons (NeuN) by double immunofluorescence staining. The results revealed that VDR predominantly colocalized with NeuN in the spinal dorsal horn, and hardly showed any overlap staining with either GFAP or Iba‐1 (Figure 2G). These findings suggest that VDR in spinal dorsal neurons may play a role in the analgesic effects of VitD_3_.

### 3.3 VitD_3_ Attenuates Ferroptosis and GABAergic Interneuron Loss in the Spinal Cord of SNI Rats

**FIGURE 3:**
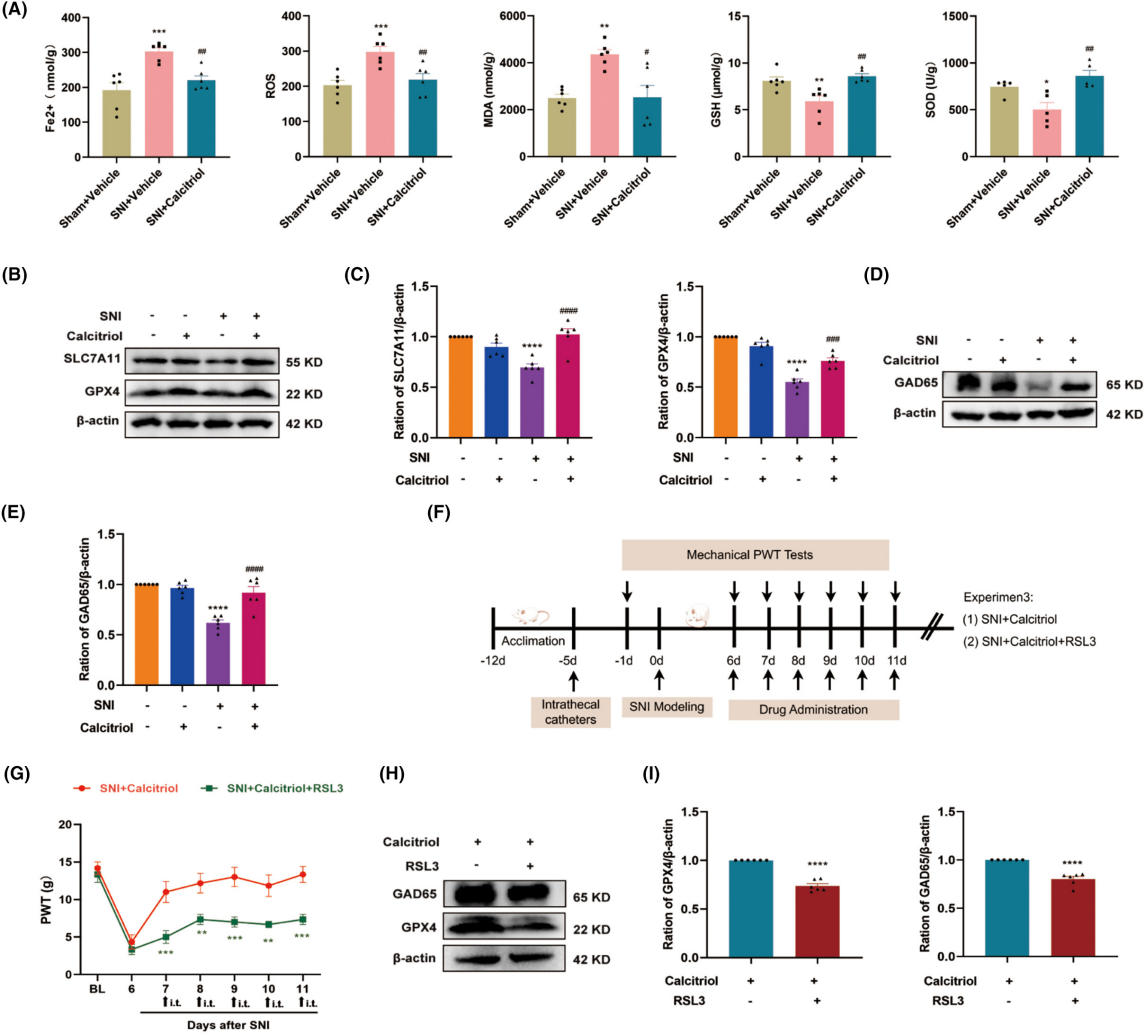
VitD3 attenuated ferroptosis and GABAergic interneuron loss in the spinal cord of SNI rats. (A) The levels of Fe2+, ROS, MDA, GSH, and SOD in the spinal cord were measured with corresponding kits (*n* = 5–6 per group). *p < 0.05, **p < 0.01, ***p < 0.001 versus Sham + Vehicle group, ^#^ *p* < 0.05, ^##^ *p* < 0.01 versus SNI + Vehicle group. (B, C) Representative blots and quantification of SLC7A11 and GPX4 in the spinal cord of different groups were presented (*n* = 6 per group). ****p < 0.0001 versus the Sham + Vehicle group, ^###^ *p* < 0.001, ^####^ *p* < 0.0001 versus the SNI + Vehicle group. (D, E) Representative blots and quantification of GAD65 in the spinal cord of different groups were presented (*n* = 6 per group). ****p < 0.0001 versus the Sham + Vehicle group, ^####^ *p* < 0.0001 versus the SNI + Vehicle group. (F) The timeline of experimental procedures. (G) Rats were intrathecally administered with calcitriol (1 μg) alone or co‐application with RSL3 (5 μg) once daily from Day 7 to Day 11 following SNI. Mechanical allodynia was conducted at 2 h after daily treatment, **p < 0.01, ***p < 0.001 versus SNI + Calcitriol group, *n* = 6 per group. (H, I) Representative western blot bands and quantification of GAD65 and GPX4 in the spinal cord of SNI + Calcitriol and SNI + Calcitriol+RSL3 group were presented. ****p < 0.0001 < 0.0001 versus SNI + Calcitriol group, *n* = 6 per group.

To investigate whether VitD_3_ could inhibit the enhanced ferroptosis induced by SNI, we assessed the changes in ferroptosis‐related indicators in the spinal cord. As illustrated in Figure 3A, treatment with calcitriol significantly alleviated the increased level of Fe^2+^, ROS, and MDA in the spinal cord compared with the SNI + Vehicle group. Meanwhile, the suppressed activity of GSH and SOD was reversed by calcitriol treatment. Furthermore, western blot analysis revealed that calcitriol treatment significantly hindered the SNI‐induced downregulation of GPX4 and SLC7A11 (Figure 3B,C). These data support the resistant role of VitD_3_ in ferroptosis under neuropathic pain conditions.

Considering the neuroprotective effect of VitD_3_ in neurodegenerative disease [22], we investigated whether VitD_3_ could reduce SNI‐induced neuronal injury, particularly the GABAergic interneurons in the spinal cord. Glutamic acid decarboxylase 65 (GAD65) is a GABA‐synthesizing enzyme that can be used to indicate the number and functional changes of GABA interneurons [35]. Consequently, we observed a statistically significant decrease in the protein levels of GAD65 in the spinal cord following SNI, and treatment with calcitriol markedly enhanced the expression of GAD65 (Figure 3D,E). To deeply explore whether the protective effect on GABA neurons was attributed to the inhibition of ferroptosis by calcitriol, the ferroptosis inducer RSL3 (5 μg) combined with calcitriol was intrathecally injected into SNI rats and then measured the pain behavior. The timeline of this test is displayed in Figure 3F. As shown in Figure 3G, the behavioral test showed that the RSL3 treatment partially abolished the antinociceptive effect of calcitriol. Moreover, western blot results revealed that administration of RSL3 significantly hindered the upregulation of GAD65 and GPX4 induced by calcitriol treatment (Figure 3H,I). These data hint that VitD_3_ protects the spinal GABAergic interneuron against ferroptosis, thereby attenuating neuropathic pain.

### 3.4 VitD_3_ Mitigates SNI‐Induced Mitochondrial Dysfunction in the Spinal Cord

**FIGURE 4:**
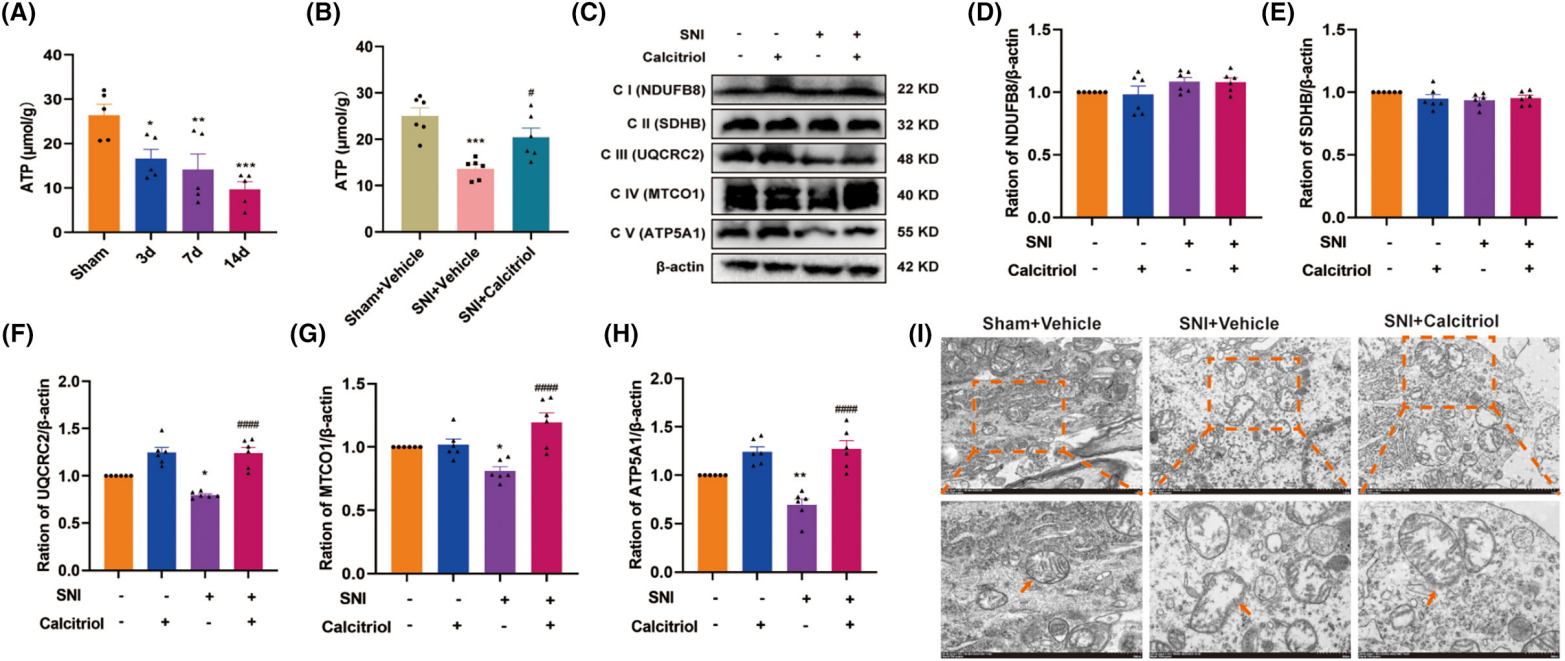
VitD_3_ mitigated SNI‐induced mitochondrial dysfunction in the spinal cord. (A, B) The content of ATP in the spinal cord was measured. (C–H) Representative western blot bands of mitochondrial ETC complexes (NDUFB8, SDHB, UQCRC2, MTCO1, and ATP5F1A) in the spinal cord of different groups were presented. Quantitative analysis of scanning densitometry was performed. *p < 0.05, **p < 0.01 versus Sham + Vehicle group, ^####^ *p* < 0.0001 versus SNI + Vehicle group, *n* = 4–6 in each group. (I) Representative electron morphological changes in the mitochondrial in the spinal cord among groups. The labeled yellow boxes highlight the mitochondrial ultrastructure. scale bar: 1 μm and 500 nm, *n* = 3 in each group.

Mitochondrial metabolism dysfunction participates in governing ferroptosis and is implicated in the pathogenesis of neuropathic pain [36, 37]. Here, we evaluated the effect of VitD_3_ on the activity of mitochondrial oxidative metabolism in the spinal cord. Initially, we measured the ATP content in the spinal cord of sham and SNI rats. We observed that the levels of ATP were time‐dependently declined from Day 3 to Day 14 following SNI, a phenomenon that was reversed by calcitriol treatment (Figure 4A,B). In the mitochondrial metabolic pathway, the mitochondrial ETC consists of five protein complexes and plays a pivotal role in ATP production. Subsequently, we analyzed the protein levels of the five mitochondrial ETC complex subunits. As shown in Figure 4C–H, western blot results showed that calcitriol treatment significantly restored the SNI‐induced downregulation of ETC Complex III, IV, and V (UQCRC2, MTCO1, and ATP5F1A), as compared to the SNI + Vehicle group. However, the protein levels of Complex I and II (NDUFB8 and SDHB) unexpectedly didn't meet statistical differences. Furthermore, the mitochondrial ultrastructure in spinal neurons by using TEM exhibited swollen mitochondria, cristae rupture, and greater membrane density in response to SNI, which were reversed by calcitriol treatment (Figure 4I). These findings indicate that VitD_3_ could regulate the biological activities of mitochondria in the spinal cord.

### 3.5 PKCα/NOX4 Signaling Pathway Is Involved in the Development of SNI‐Induced Neuropathic Pain

**FIGURE 5:**
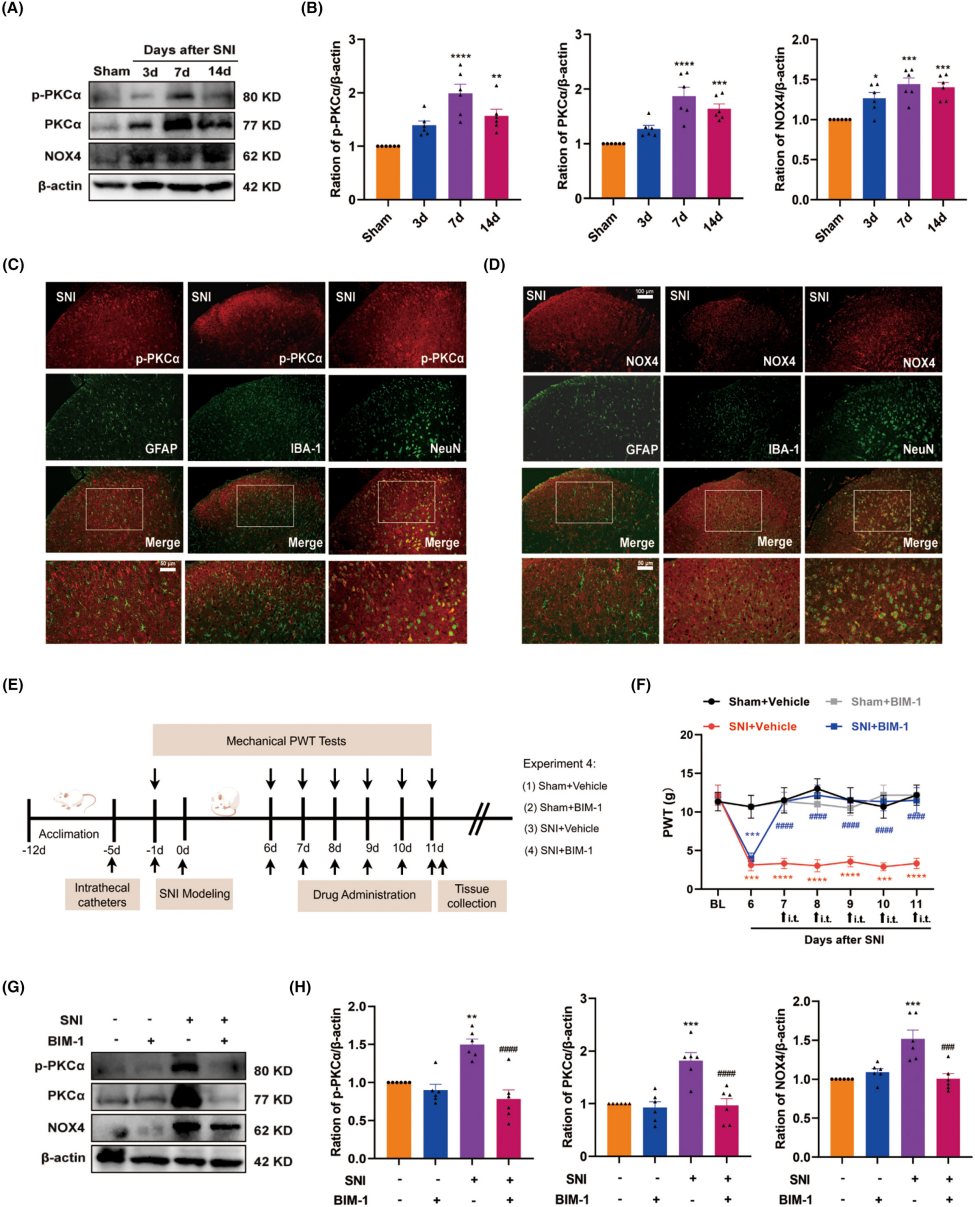
PKCα/NOX4 signaling pathway was involved in the development of SNI‐induced neuropathic pain. (A, B) Time course of p‐PKCα, PKCα, and NOX4 in the spinal cord of after SNI (*n* = 6 per group). *p < 0.05, **p < 0.01, ***p < 0.001, ****p < 0.0001 versus sham group. (C, D) Double immunofluorescence of p‐PKCα and NOX4 with GFAP, IBA1, and NeuN in the spinal dorsal horn of SNI rats (*n* = 4 per group). The white boxes indicated typical co‐staining cells. Scale bar: 100 and 50 μm. (E) The time schedule of the present experiment. (F) BIM‐1 (100 μM, i.t.) was given once daily from Day 7 to Day 11 following SNI. Mechanical allodynia was performed at 1.5 h after daily treatment. ***p < 0.001, ****p < 0.0001 versus Sham + Vehicle group, ^####^ *p* < 0.000 versus SNI + Vehicle group. (G, H) Representative western blot bands and quantification of J p‐PKCα, PKCα, and NOX4 in the spinal cord among groups, **p < 0.01, ***p < 0.001 versus the sham group, ^###^ *p* < 0.001, ^####^ *p* < 0.0001 versus the SNI + Vehicle group, *n* = 6 in each group.

It was previously reported that PKCα contributed to pain hypersensitivity in chronic inflammatory pain [38]. Moreover, NOX4 is a downstream effector of PKCα that generates ROS and is also involved in pain processing [39]. Accordingly, we examined the levels of p‐PKCα, PKCα, and NOX4 in the spinal cord by western blot. As illustrated in Figure 5A,B, compared with the sham control, the protein expression of p‐PKCα, PKCα, and NOX4 consistently increased in the spinal cord from Day 3 to Day 14, peaking on Day 7. Additionally, double immunostaining showed that both p‐PKCα and NOX4 were predominately colocalized with neurons in the spinal dorsal horn, but rarely with astrocytes or microglia (Figure 5C,D). These results suggest that the PKCα/NOX4 signaling pathway may function in spinal dorsal neurons in pain processing.

To confirm the role of the PKCα/NOX4 signaling pathway in SNI‐induced neuropathic pain, we applied a pharmacological approach using the broad‐range PKCα inhibitor BIM‐1. The timeline of this test is depicted in Figure 5E. BIM‐1(100 μM) was intrathecally injected for 5 consecutive days starting from Day 7 after SNI, and the behavioral test was conducted before and 1.5 h after BIM‐1 injection. As illustrated in Figure 5F, the behavioral test showed that the PWT was significantly increased in BIM‐1‐treated rats compared with the vehicle‐treated rats. Importantly, administration of BIM‐1 markedly reduced the protein expression of p‐PKCα, PKCα, and NOX4 in the spinal cord (Figure 5G,H). These results suggest that the spinal PKCα/NOX4 signaling pathway plays a functional role in neuropathic pain.

### 3.6 Pharmacological Inhibition of PKCα/NOX4 Signaling Pathway Suppressed Ferroptosis and Mitochondrial Dysfunction

**FIGURE 6:**
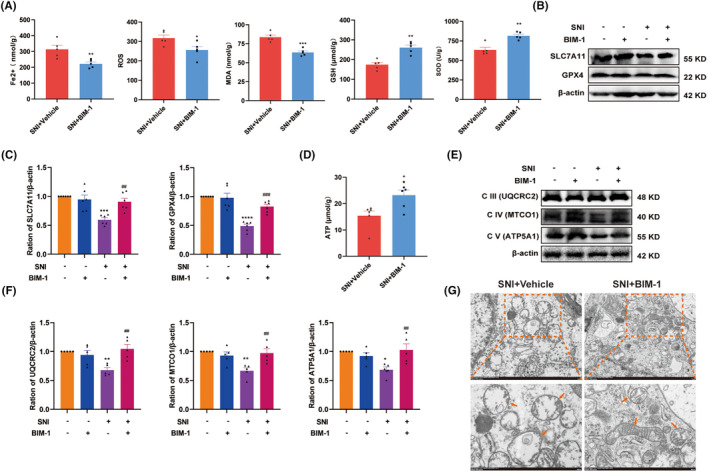
Pharmacological inhibition of PKCα/NOX4 signaling pathway suppressed ferroptosis and mitochondrial dysfunction. (A) The levels of Fe2+, ROS, MDA, GSH, and SOD in the spinal cord of SNI + Vehicle and SNI + BIM‐1 groups were measured (*n* = 5–6 per group). *p < 0.05, **p < 0.01, ***p < 0.001 versus SNI + Vehicle group. (B, C) Representative blots and quantification of SLC7A11 and GPX4 in the spinal cord of different groups were presented (*n* = 5–6 per group). ***p < 0.001, ****p < 0.0001 versus sham group, ^##^ *p* < 0.01, ^###^ *p* < 0.00 versus SNI + Vehicle group. (D) The levels of ATP in spinal cord of SNI + Vehicle and SNI + BIM‐1 groups were measured (*n* = 6 per group). *p < 0.05 versus SNI + Vehicle group. (E, F) Representative western blot bands of mitochondrial ETC Complex III‐V (UQCRC2, MTCO1, and ATP5F1A) in the spinal cord of different groups were presented. Quantitative analysis of scanning densitometry was performed. *p < 0.05, **p < 0.01 versus Sham + Vehicle group, ^#^ *p* < 0.05, ^##^ *p* < 0.01 versus SNI + Vehicle group, *n* = 5 in each group. (G) Representative electron morphological changes of the mitochondrial in the spinal cord of SNI + Vehicle and SNI + BIM‐1 groups (*n* = 3 per group). The labeled yellow boxes highlight the mitochondrial ultrastructure. scale bar: 1 μm and 500 nm.

Next, to test whether the PKCα/NOX4 signaling pathway contributed to SNI‐induced ferroptosis and mitochondrial dysfunction, we assessed the changes in ferroptosis‐related indexes in the spinal cord after intrathecal injection with BIM‐1. As shown in Figure 6A, compared with the vehicle‐treated group, the level of Fe^2+^, ROS, and MDA were significantly decreased by BIM‐1 treatment, while the activity of GSH and SOD were increased. In response to BIM‐1 treatment, higher levels of GPX4 and SLC7A11 in the spinal cord were observed in SNI rats (Figure 6B,C). Simultaneously, BIM‐1 treatment significantly attenuated SNI‐caused decline in ATP contents (Figure 6D). As expected, western blot results showed that BIM‐1 administration significantly increased the levels of ETC Complex III, IV, and V in the spinal cord of SNI rats, as compared to the vehicle‐treated group (Figure 6E,F). The influence of BIM‐1 on mitochondrial morphology was also evaluated, and the mitochondrial morphological injury in spinal neurons caused by SNI was alleviated by BIM‐1 treatment (Figure 6G). These results suggest that SNI‐triggered ferroptosis and mitochondrial dysfunction in the spinal cord probably in a PKCα/NOX4 signaling pathway‐dependent manner.

### 3.7 VitD_3_ Alleviates Pain Hypersensitivity via Inhibiting PKCα/NOX4 Signaling Pathway

**FIGURE 7:**
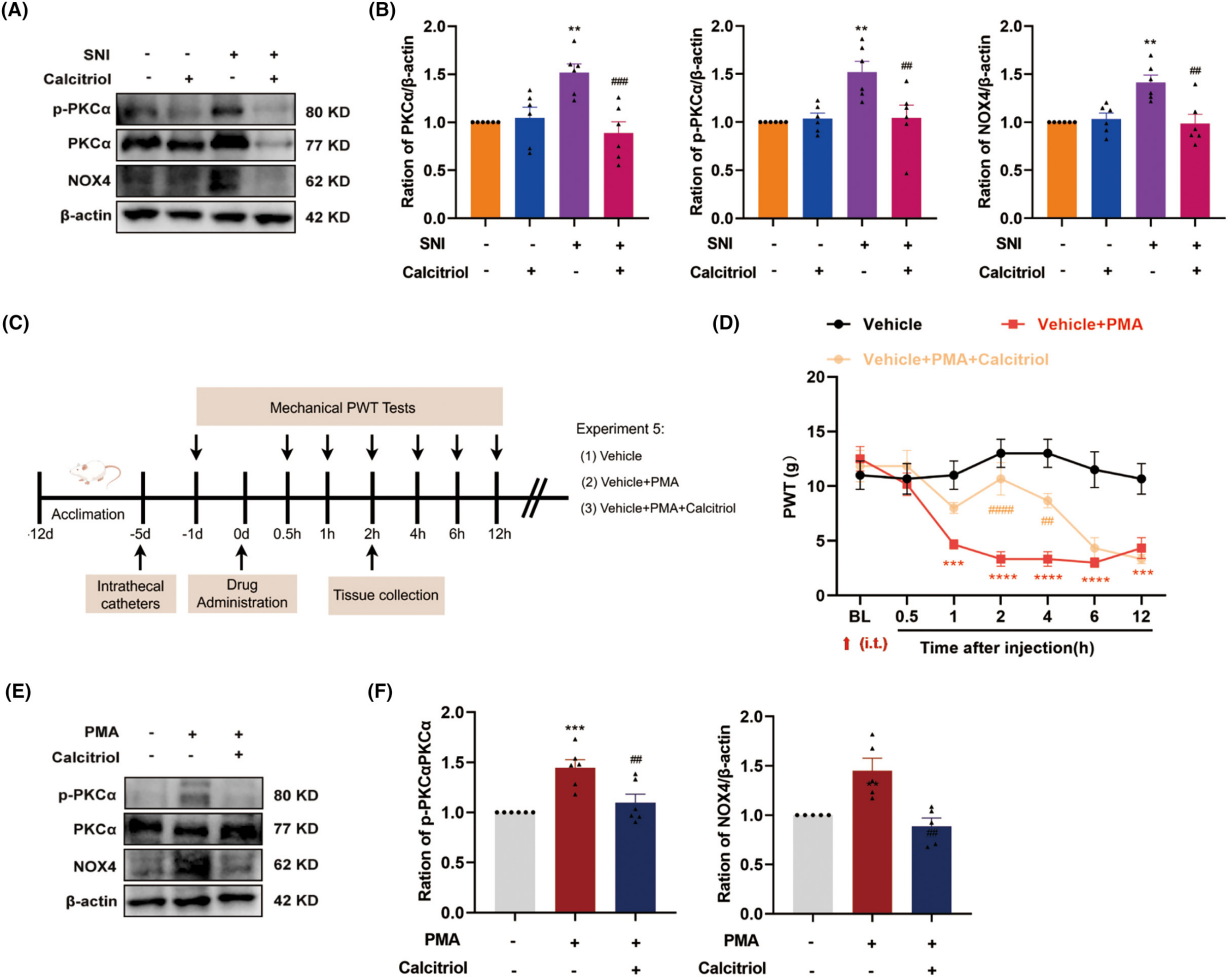
VitD3 alleviated pain hypersensitivity via inhibiting PKCα/NOX4 signaling pathway. (A, B) Representative western blot bands and quantification of p‐PKCα, PKCα, and NOX4 in the spinal cords of animals from different groups were presented. **p < 0.01 versus Sham + Vehicle group, ^##^ *p* < 0.01, ^###^ *p* < 0.001 versus SNI + Vehicle group, *n* = 6 in each group. (C) Time schedule of the experiment. (D) Naïve rats were intrathecally injected with a single dose of PMA (2.5 μg) alone or in combination with calcitriol (1 μg). Mechanical allodynia was measured before injection and 0.5, 1, 2, 4, 6, and 12 h after injection. ***p < 0.001, ****p < 0.0001 compared with Vehicle‐treated rats, ^##^ *p* < 0.01, ^####^ *p* < 0.0001 compared with PMA‐treated rats, *n* = 6 in each group. (E, F) Representative western blot bands and quantification of p‐PKCα and NOX4 in the spinal cords of animals from different groups were presented. The phosphorylation levels of PKCα were normalized to the total protein levels. **p < 0.01, ***p < 0.001 compared with Vehicle‐treated rats, ^##^ *p* < 0.01 compared with PMA‐treated rats, *n* = 4–6 in each group.

The molecular basis for the analgesic effect of VitD_3_ remains to be elucidated. Based on our above findings, we further explored the role of the PKCα/NOX4 signaling pathway in VitD_3_‐mediated antinociception. As a result, western blot results displayed that calcitriol treatment significantly reduced the protein levels of p‐PKCα, PKCα, and NOX4 in the spinal cord, as compared to the vehicle‐treated SNI group (Figure 7A,B). To further verify the potential relationship between the PKCα/NOX4 signaling pathway and VitD_3_ in nociception, we treated naïve rats with the PKCα activator PMA alone or in combination with calcitriol and analyzed the changes in pain behavior. The schedule for the drug delivery and testing is shown in Figure 7C. PMA (2.5 μg) and calcitriol were intrathecally given in a single dose, and the PWT was measured before the injection and 0.5–12 h after the injection. As illustrated in Figure 7D, compared with the vehicle group, rats that received a single dose of PMA exhibited a significantly decreased PWT from 1 h after injection, and lasted at least to 12 h. Moreover, calcitriol treatment attenuated the mechanical allodynia induced by PMA administration. The analgesic effect peaked at 2 h and lasted only for 2 h. Accordingly, the spinal segments were collected at 2 h after drug administration. As expected, western blot results showed that the spinal expression of p‐PKCα/PKCα and NOX4 was significantly enhanced in response to PMA injection, which were markedly inhibited by calcitriol treatment (Figure 7E,F). Taken together, these results highlight the involvement of PKCα/NOX4 signaling pathway in the antinociceptive effect of VitD_3_.

### 3.8 VitD_3_ Preserves Spinal GABAergic Interneurons via Suppression of PKCα/NOX4 Signaling Pathway‐Mediated Ferroptosis and Mitochondrial Dysfunction

**FIGURE 8:**
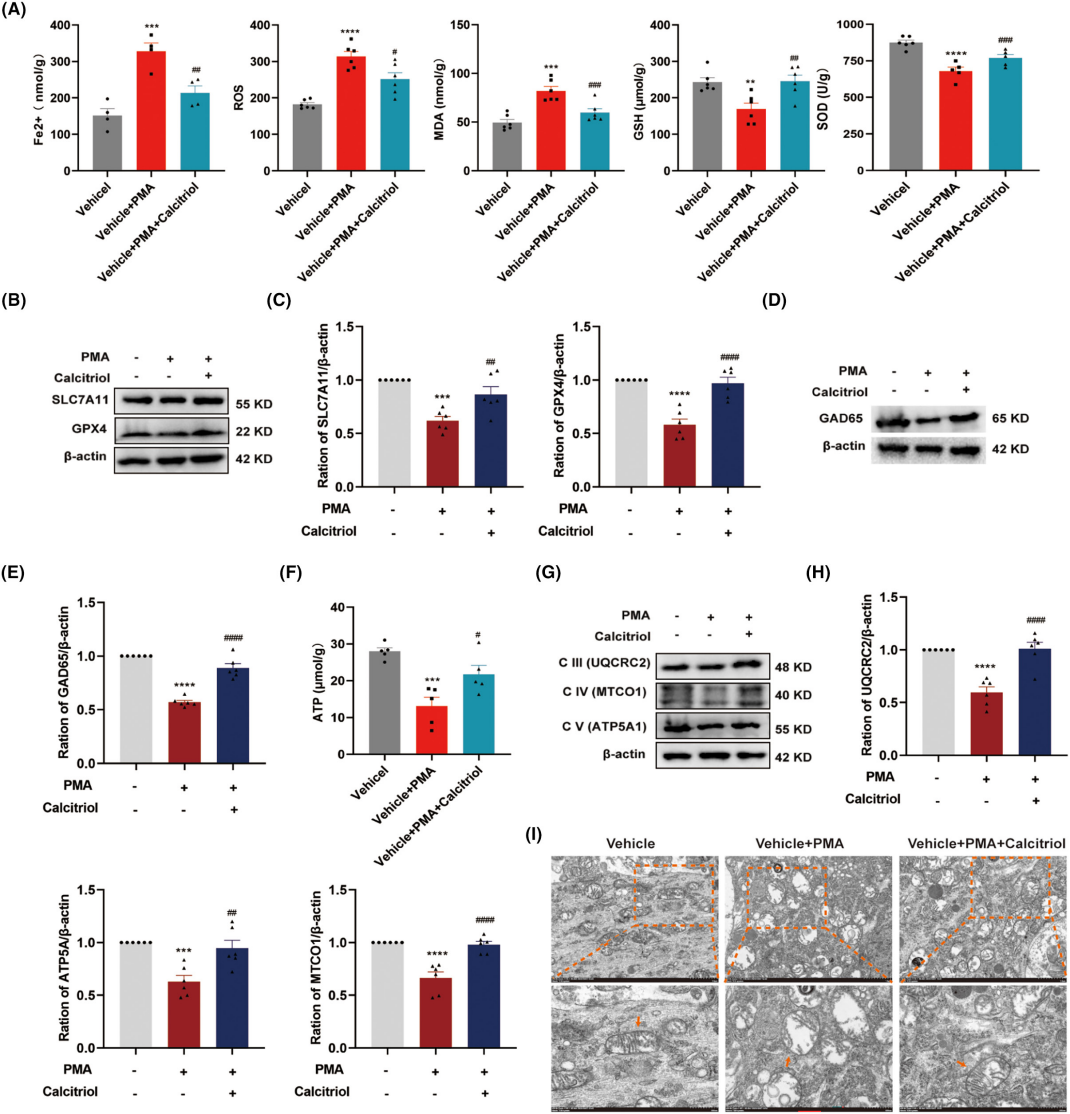
VitD_3_ preserved spinal GABAergic interneurons via suppression of PKCα/NOX4 signaling pathway‐mediated ferroptosis and mitochondrial dysfunction. (A) The levels of Fe2+, ROS, MDA, GSH, and SOD in the spinal cord in different groups were assessed (*n* = 4–6 per group). **p < 0.01, ***p < 0.001, ****p < 0.0001 compared with Vehicle‐treated rats, ^#^ *p* < 0.05, ^##^ *p <* 0.01, ^###^ *p* < 0.001 compared with PMA‐treated rats. (B, C) Representative western blot bands and quantification of SLC7A11 and GPX4 in the spinal cords of different groups were presented (*n* = 6 per group). ***p < 0.001, ****p < 0.0001 compared with Vehicle‐treated rats, ^##^ *p* < 0.01, ^####^ *p* < 0.0001 compared with PMA‐treated rats. (D, E) Representative western blot bands and quantification of GAD65 in the spinal cords of different groups were presented (*n* = 6 per group). ****p < 0.0001 compared with Vehicle‐treated rats, ^####^ *p* < 0.0001 compared with PMA‐treated rats. (F) The levels of ATP in spinal cord were measured (*n* = 5 per group). ***p < 0.001 compared with Vehicle‐treated rats, ^#^ *p* < 0.05 compared with PMA‐treated rats. (G, H) Representative western blot bands of mitochondrial ETC Complex III‐V (UQCRC2, MTCO1, and ATP5F1A) in the spinal cord of different groups were presented. ***p < 0.001, ****p < 0.0001 compared with Vehicle‐treated rats, ^##^ *p* < 0.01, ^####^ *p* < 0.0001 compared with PMA‐treated rats, *n* = 5–6 in each group. (I) Representative morphological changes in the mitochondrial in the spinal cord (*n* = 3 per group). The labeled yellow boxes highlight the mitochondrial ultrastructure. scale bar: 1 μm and 500 nm.

To ascertain whether VitD_3_ suppressed pain behaviors induced by PKCα/NOX4 pathway activation in naïve rats involves a similar mechanism in SNI rats, we further examined the expression of ferroptosis markers and GAD65 in the spinal cord. Surprisingly, PMA administration induced a dramatic elevation in Fe^2+^, ROS, and MDA, and a decline in GSH and SOD in the spinal cord, all of which were reversed by calcitriol treatment (Figure 8A). Additionally, calcitriol treatment significantly alleviated PMA‐induced downregulation of spinal SLC7A11 and GPX4, as well as GAD65 (Figure 8B–E). Furthermore, the effects of VitD_3_ on PKCα/NOX4 pathway‐mediated mitochondrial function were also explored. As shown in Figure 8F, the ATP content was significantly decreased after PMA administration compared with the vehicle group. Intriguingly, calcitriol treatment significantly restored PMA‐induced downregulation of ATP. Simultaneously, the protein levels of ETC Complex III, IV, and V were obviously decreased in the spinal cord of PMA‐treated rats, which were dramatically alleviated by calcitriol treatment (Figure 8G,H). Furthermore, TEM results showed swollen mitochondria with reduction in cristae and rupture of the outer membrane in spinal cord neurons after PMA administration (Figure 8I). However, calcitriol treatment attenuated these mitochondrial damages. These findings suggest that the protective effect of VitD_3_ on spinal GABAergic interneurons through the suppression of PKCα/NOX4 signaling pathway‐mediated ferroptosis and mitochondrial dysfunction.

## 4 Discussion

Albeit the understanding of the mechanism underlying neuropathic pain has been deepened over the past decades, the first‐line pharmaceutical therapy for neuropathic pain often has limited efficacy and non‐negligible side effects [3]. Interest in Vitamin D as an alternative therapeutic approach for neuropathic pain has recently increased dramatically [40, 41]. However, the underlying mechanism of the action remains unclear. In the present study, we showed that intrathecal administration of VitD_3_ alleviated mechanical allodynia by preventing the loss of GABAergic interneurons in the spinal cord. This effect may be related, at least in part, to the inhibition of mitochondrial‐associated ferroptosis mediated by the PKCα/NOX4 signaling pathway, in a VDR‐activated manner. These results provided new theoretical evidence and a novel perspective regarding the utilization of VitD_3_ for treating neuropathic pain.

Ferroptosis is a unique modality of regulated cell death that distinct from apoptosis and necroptosis, characterized by lipid peroxidation initiated by iron overload and ROS generation [9]. Iron overload facilitates excessive ROS production via the Fenton reaction, which interacts with polyunsaturated fatty acids (PUFAs) of lipid membranes and induce lipid peroxidation, and eventually leads to ferroptosis‐like cell death [42]. Additionally, inactivation of GPX4 by GSH depletion promotes ROS accumulation from lipid peroxidation [43]. Accumulating evidence has ascribed an essential pathophysiological role for ferroptosis‐like cell death in the spinal cord within various models of neuropathic pain [12]. Notably, intraperitoneal administration of ferroptosis inhibitors has been shown to alleviates pain‐like behaviors in rat models of chronic constriction injury (CCI)‐induced neuropathic pain [10, 11]. Consistently, data from our study showed an overwhelming accumulation of Fe^2+^, ROS, and MDA in the spinal cord of SNI rats. Meanwhile, we also observed decreased activity of GSH and SOD, as well as a decrease in protein levels of SLC7A11 and GPX4 in the spinal cord following SNI. These data support the essential role of ferroptosis in the development of neuropathic pain, suggesting that ferroptosis as a viable novel therapeutic target for neuropathic pain treatment.

Vitamin D is currently appealing growing attention across disciplines because of its diverse biological features, encompassing anti‐inflammatory, antioxidative, and neuroprotective effects [21, 44]. The biological functions of vitamin D are achieved by VitD_3_ through binding with the VDR, a nuclear receptor that is widely expressed in the brain and spinal cord [34]. In the present study, we demonstrated that intrathecal administration of calcitriol (1 μg, or 2 μg) had an analgesic effect in rats with neuropathic pain in a dosage‐dependent manner. Moreover, the protein levels of VDR were found decreased in the spinal cord following SNI, which could be reversed by calcitriol treatment. Our results also indicated that VDR was specifically localized in the spinal dorsal horn neurons of SNI rats. Recently, vitamin D/VDR signaling has been reported to regulate ferroptosis by modulating signal transduction in a cell‐specific manner. For example, Hu et al. showed that VDR activation by paricalcitol attenuated cisplatin‐induced renal injury by inhibiting ferroptosis via trans‐regulation of GPX4 [45]. Similarly, Xu et al. reported that VitD_3_ reduced osteoblast ferroptosis by activating the nuclear factor erythroid 2–related factor 2 (NRF2)/GPX4 signaling pathway through a mechanism mediated by VDR [46]. Consistently, our study demonstrated that treatment with calcitriol effectively alleviated SNI‐induced ferroptosis in the spinal cord.

It is generally acknowledged that the GABAergic system participates in the transmission of nociceptive information in the spinal cord. Of note, GABAergic interneurons exhibit a high metabolic requirement, rendering them more vulnerable to redox dysregulation and oxidative stress [47]. Spinal GABAergic interneurons compromised by ferroptosis have been reported in a mouse model of bone cancer pain showing that ferroptosis inhibition prevented the loss of spinal GAD65^+^ interneurons [6]. Herein, we found that SNI‐induced downregulation of GAD65 in the spinal cord was rescued by calcitriol treatment, suggesting that VitD_3_ alleviates neuropathic pain by protecting spinal GABAergic interneurons from ferroptotic cell death. To further validating the role of ferroptosis in GABAergic interneurons loss, we employed the ferroptosis agonist RSL3 to assess whether it could reverse the protective effects of calcitriol in SNI rats. Intriguingly, the improved GAD65 and antinociceptive effect induced by calcitriol were abolished by RSL3, which triggers ferroptosis by inhibition of GPX4 enzyme's function. Cumulatively, these observations indicate that the analgesic effect of VitD_3_ might be related to its protective effects on GABAergic interneurons by inhibiting ferroptosis.

Mitochondria play a crucial and proactive role in ferroptotic cell death through its oxidative metabolism function [14]. Excessive intracellular ROS can damage mitochondrial function by intruding mitochondrial DNA and ETC complexes, and therefore promoting ferroptosis [48]. Mounting evidence suggests that vitamin D/VDR is crucial for mitochondrial respiration and oxidative capacity, thus involved in essential bioenergetic metabolic pathways [49, 50]. Loss of VDR function has been shown to decrease mitochondrial respiration and ATP production from oxidative phosphorylation [50]. Additionally, it has been found that vitamin D supplementation improved mitochondrial biogenesis in skeletal muscle by increasing the activity and protein expression of mitochondrial ETC complexes through a nuclear mechanism by VDR [51]. In this regard, we explored the functional and morphological relevance of mitochondria in ferroptosis after VitD_3_ treatment. Experiments from the current study observed decreased levels of ETC Complex III‐V and ATP production, as well as aberrant mitochondrial morphology in the spinal cord of SNI rats. Importantly, calcitriol treatment attenuated these damages. These results indicate that the anti‐ferroptosis of VitD_3_ is closely associated with its regulation of mitochondrial function.

**FIGURE 9:**
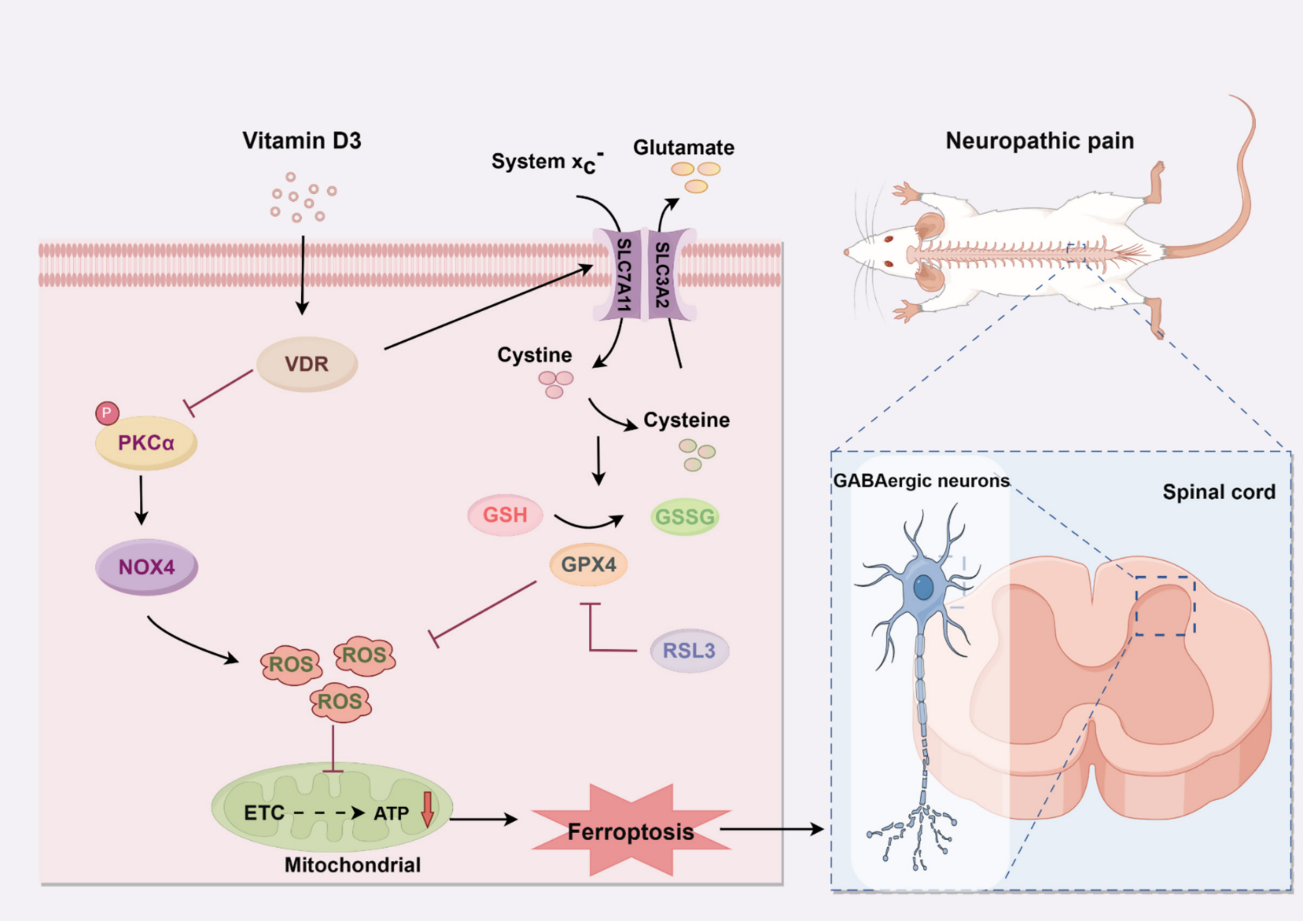
Schematic diagram of the analgesic mechanism of vitamin D_3_ in neuropathic pain.

Although the precise molecular mechanisms governing mitochondria‐associated ferroptosis by VitD_3_ in neuropathic pain are not fully understood, NOX4, a key dominator of ROS production, has been shown to promote ferroptosis through the impairment of mitochondrial oxidative metabolism [37]. The Nrf2/heme oxygenase‐1 (HO‐1) signaling pathway plays a critical role in GPX4‐dependent ferroptosis and mitochondrial dysfunction [52, 53]. Activation of Nrf2 has been demonstrated to inhibit ferroptosis and alleviate pain behaviors induced by SNI [54]. Park et al. reported that NOX4‐derived ROS can suppress Nrf2‐HO‐1 signaling pathway, thereby promoting ferroptosis in astrocytes [37]. Additionally, NOX4 can be activated through phosphorylation by PKCα [19]. PKCα has been shown to modulate mitochondrial proteins associated with respiratory processes [55]. Recent researches indicate that inactivation of PKCα and NOX4 can mitigate erastin‐induced ferroptosis [20, 56]. Here, we found that the protein expression of p‐PKCα, PKCα, and NOX4 was remarkably increased in the spinal cord after SNI. Double immunofluorescence results showed that the p‐PKCα and NOX4 were both colocalized mostly with neurons in the spinal dorsal horn of SNI rats. More importantly, the fact that pharmacological inhibition of PKCα significantly decreased spinal NOX4 expression, attenuated ferroptosis and mitochondria dysfunction in the spinal cord, as well as mechanical allodynia resulting from SNI. Surprisingly, our data clearly showed that calcitriol treatment significantly inhibited the expression of p‐ PKCα, PKCα, and NOX4 in the spinal cord of SNI rats. Based on the above findings, it is reasonable to hypothesize that PKCα/NOX4 signaling pathway represents a potential molecular target for VitD_3_. Accordingly, we found that activation of PKCα/NOX4 signaling by PKCα agonist in naïve rats induced nociceptive hypersensitivity, triggered increased ferroptosis, and led to a reduction of GAD65 and mitochondrial function in the spinal cord, all of which could be alleviated by calcitriol treatment. Although the mechanisms by which VitD_3_ regulated PKCα/NOX4 signaling pathway remain to be established, our present study indicates that VitD_3_ ameliorates neuropathic pain via preventing the loss of GABAergic interneurons in the spinal cord, at least partly by inhibiting mitochondria‐associated ferroptosis mediated by PKCα/NOX4 signaling pathway (Figure 9).

Nevertheless, there is a limitation to our study. We exclusively used male rats, without considering sex differences. It is widely recognized that sex differences in pain and analgesia are robust, affected by genetic, epigenetic, hormonal, and environmental factors. Sex hormones, estrogen in particular, appear to promote mitochondrial biogenesis and ROS scavenging through estrogen receptors [57]. Moreover, estrogen has been demonstrated to enhance the immunomodulatory function of vitamin D by favoring its accumulation and increasing VDR expression, resulting in a more potent anti‐inflammatory response in females than in males [58]. In this respect, future studies should be carried out to determine whether VitD_3_‐based therapeutic strategies are more effective in females than in males, paving the way for a more personalized therapeutic approach for neuropathic pain management.

## 5 Conclusions

Overall, our study for the first time concludes that VitD_3_ ameliorates neuropathic pain by preventing the loss of spinal GABAergic interneurons, which is attributed to the activation of VDR and subsequent inhibition of mitochondria‐associated ferroptosis mediated by PKCα/NOX4 signaling pathway. These findings provide a rationale based on the mechanism for the translation of VitD_3_ from bench to bedside as an adjunctive analgesic for the management of neuropathic pain.

## Conflicts of Interest

The authors declare no conflicts of interest.

## Supporting information


Data S1.

## Data Availability

The data supporting the findings of this study are available from the corresponding author upon reasonable request.
