# Supplementary material for: Vitamin D3 Attenuates Neuropathic Pain via Suppression of Mitochondria‐Associated Ferroptosis by Inhibiting PKCα/NOX4 Signaling Pathway
Source: CNS Neurosci Ther. 2024 Sep 27;30(9):e70067. doi: 10.1111/cns.70067 (PMC11427799; doi:10.1111/cns.70067)

Full unedited blot for Figure 1E

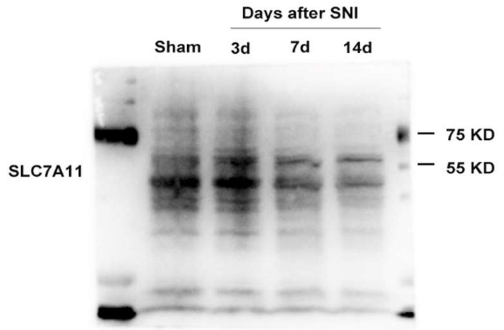

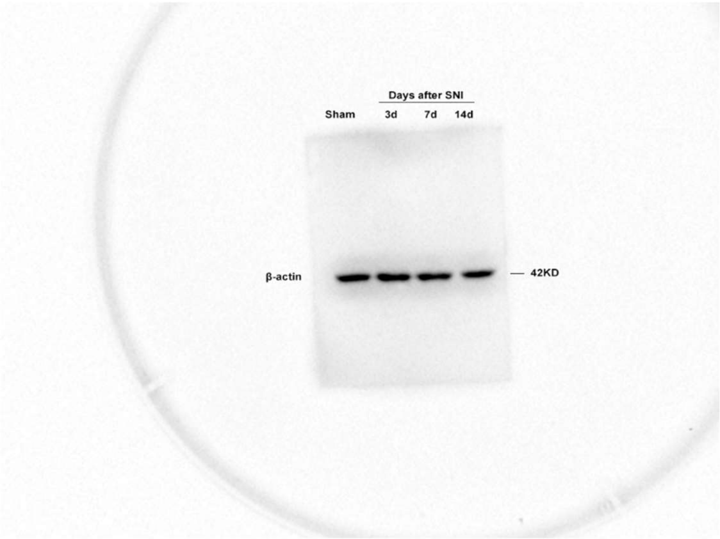

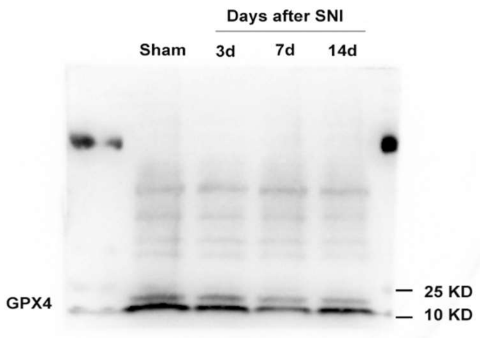

Full unedited blot for Figure 2E

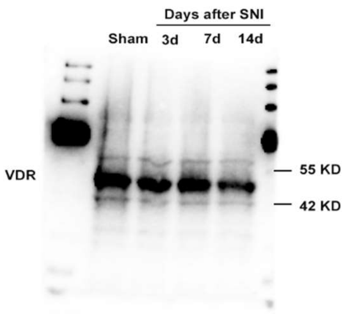

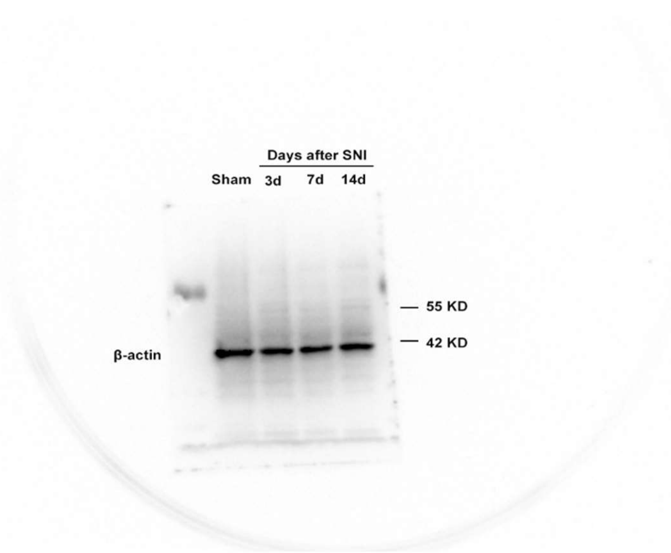

Full unedited blot for Figure 2F

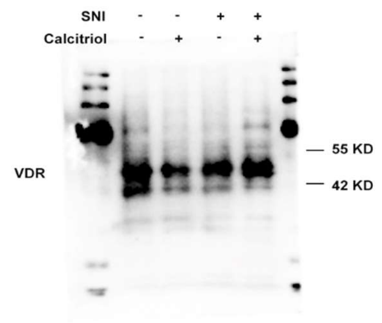

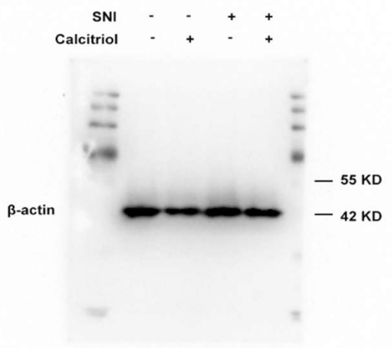

Full unedited blot for Figure 3B

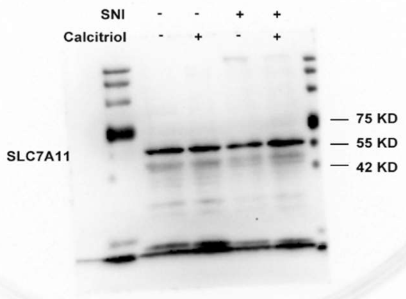

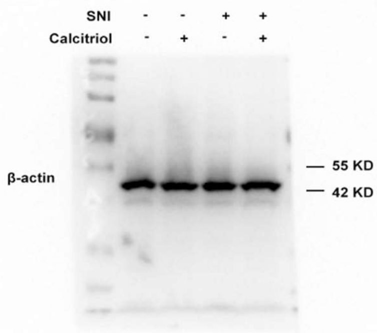

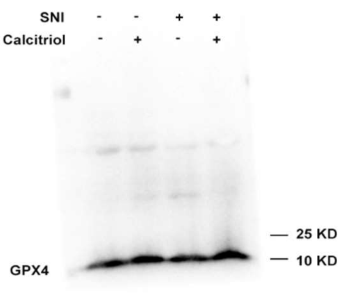

Full unedited blot for Figure 3D

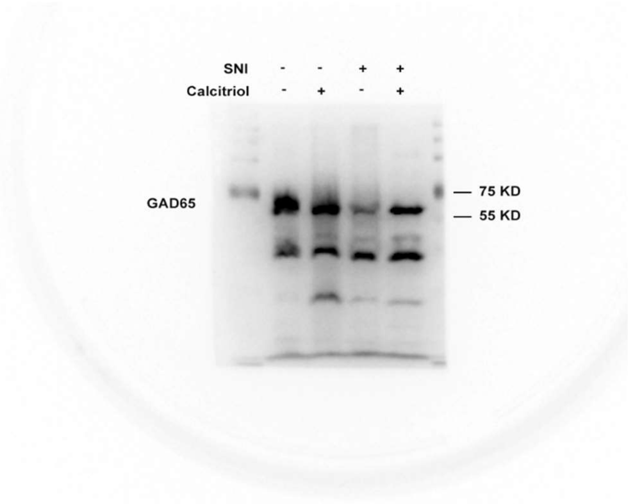

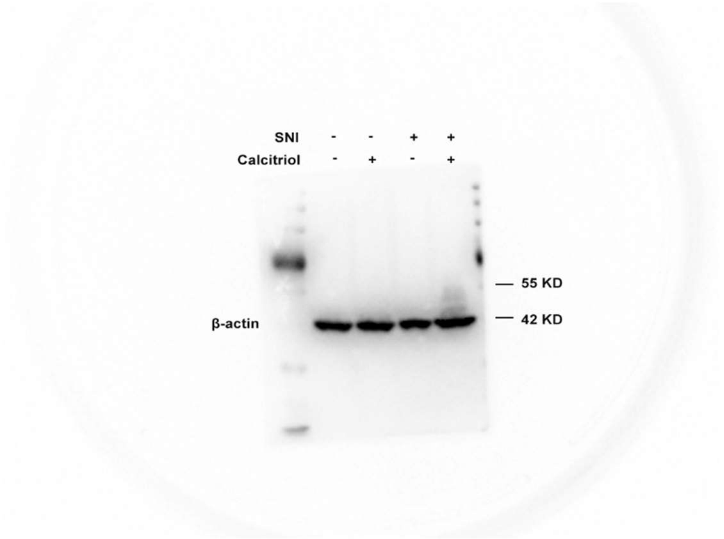

Full unedited blot for Figure 3H

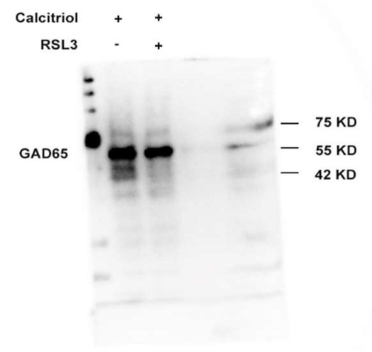

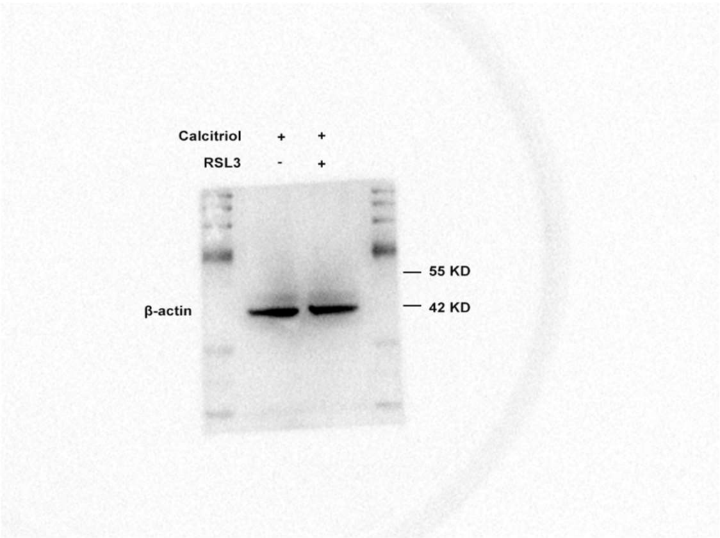

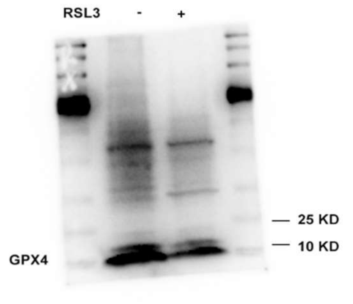

Full unedited blot for Figure 4C

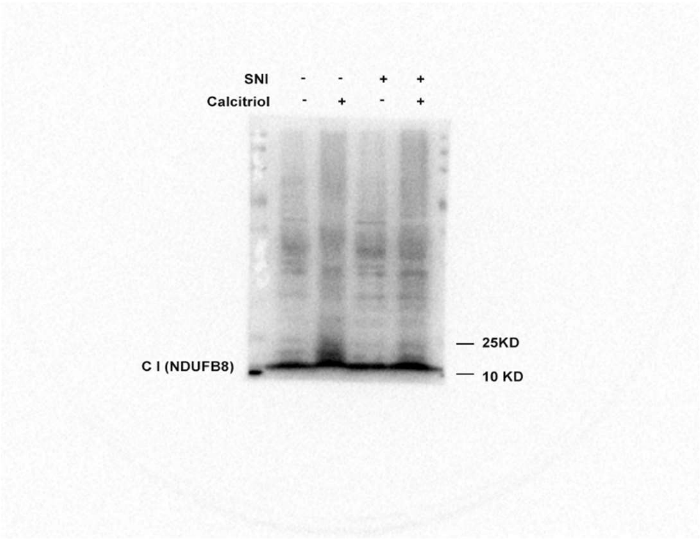

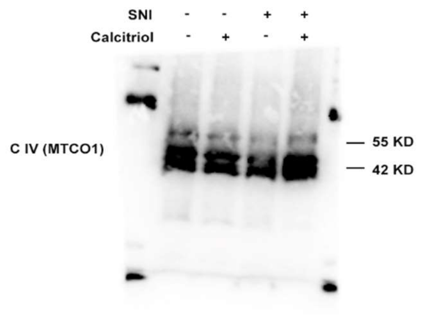

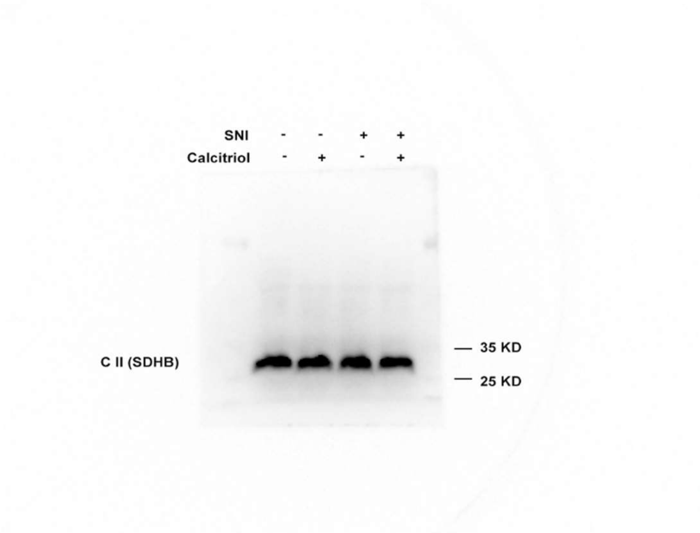

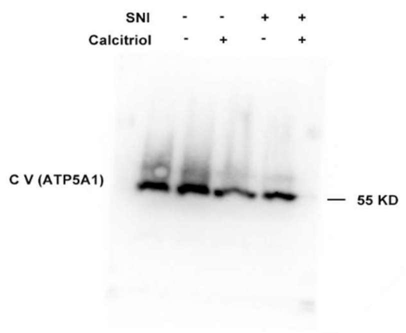

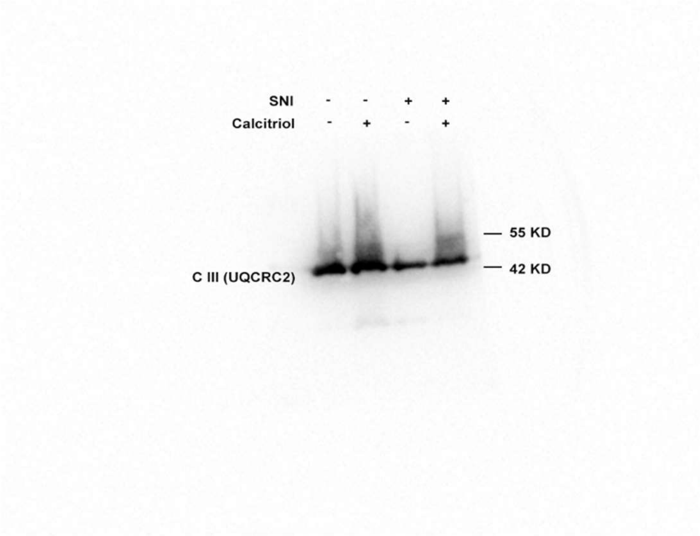

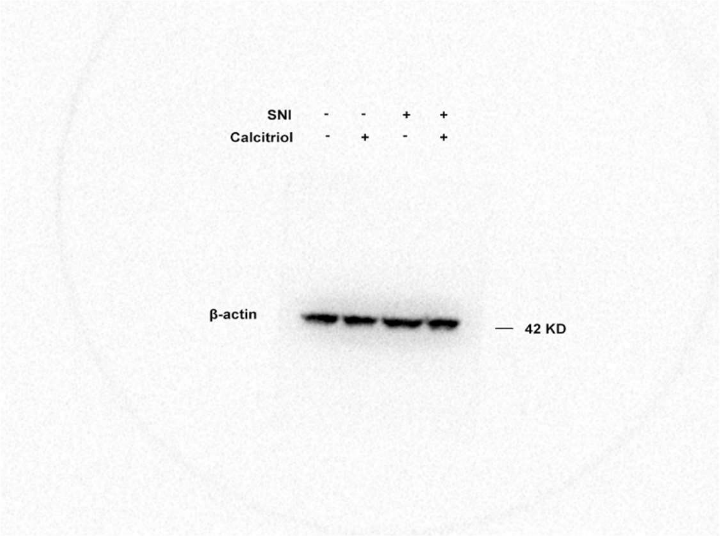

Full unedited blot for Figure 5A

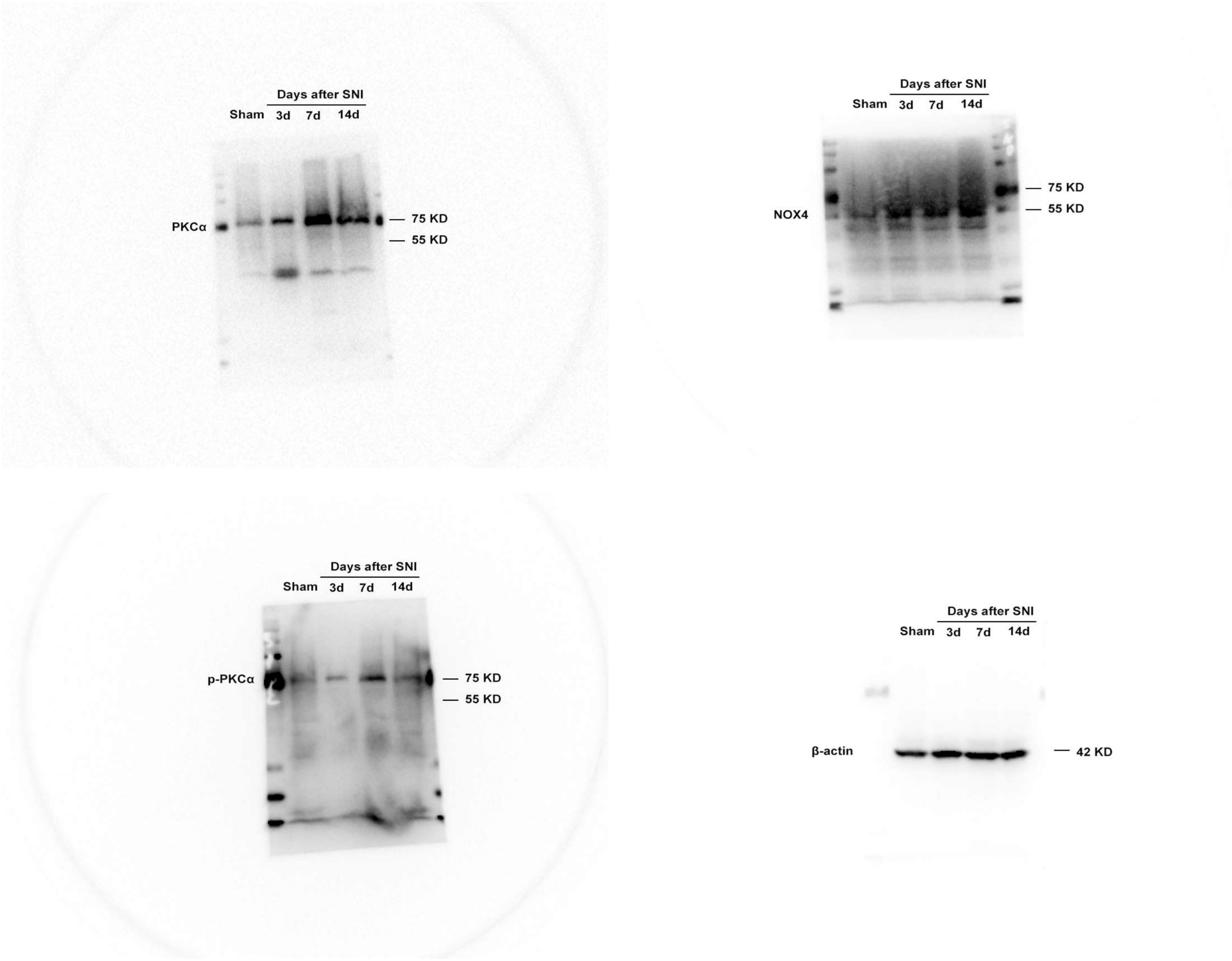

Full unedited blot for Figure 5G

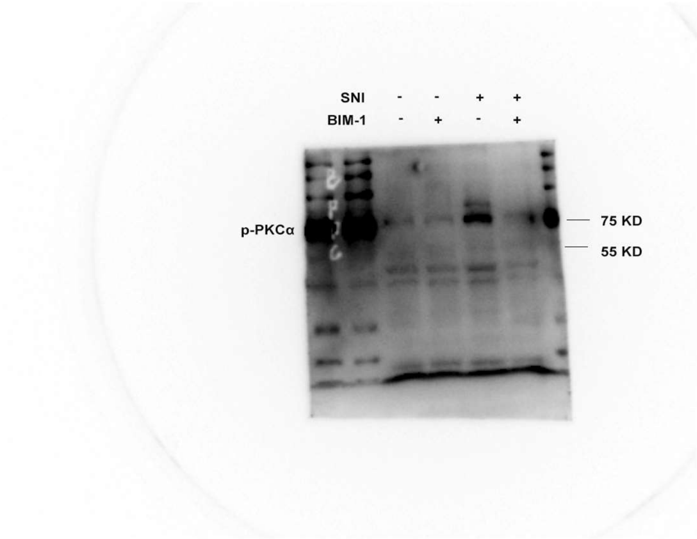

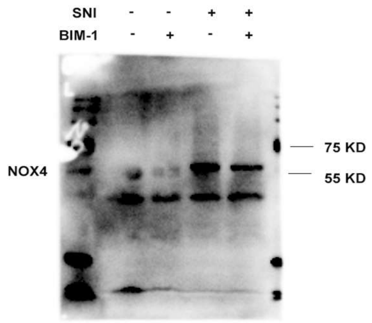

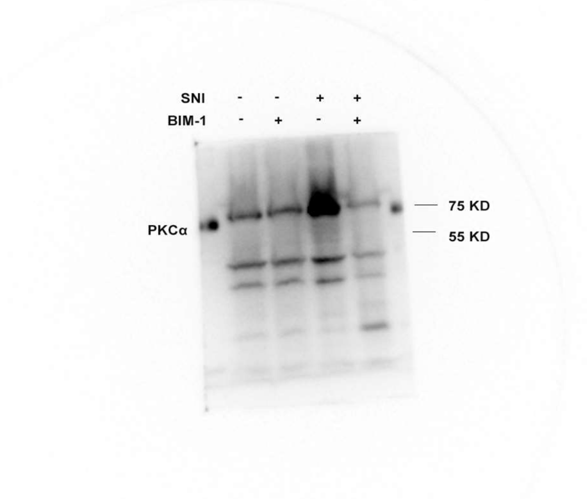

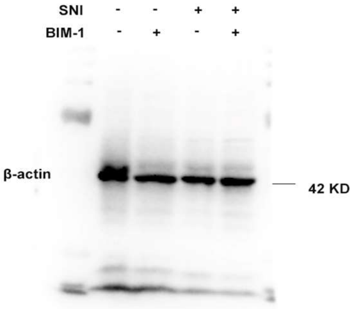

Full unedited blot for Figure 6B

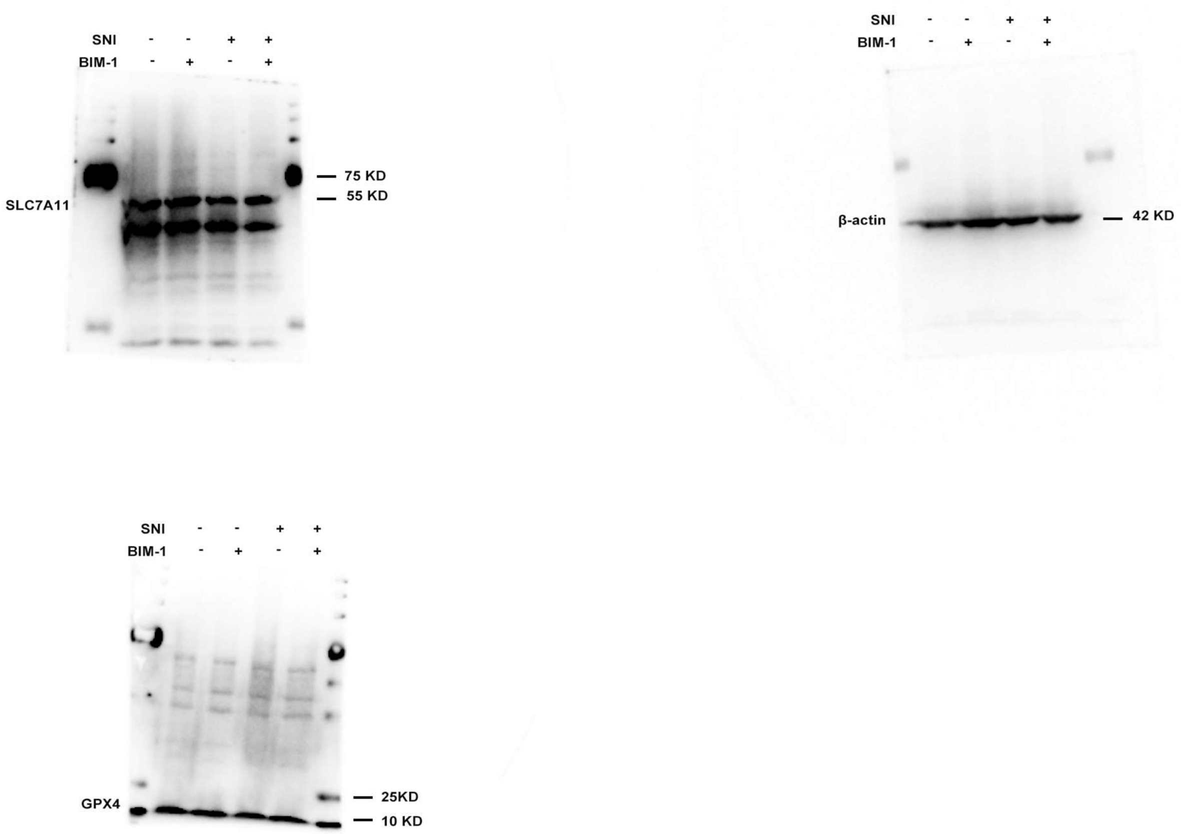

Full unedited blot for Figure 6E

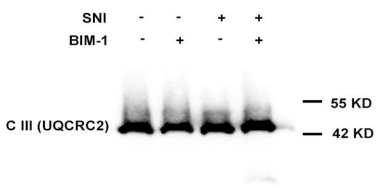

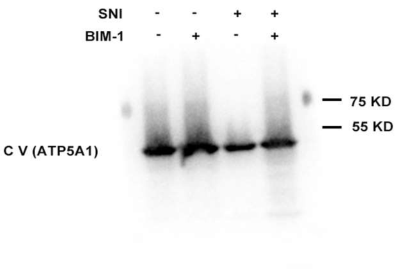

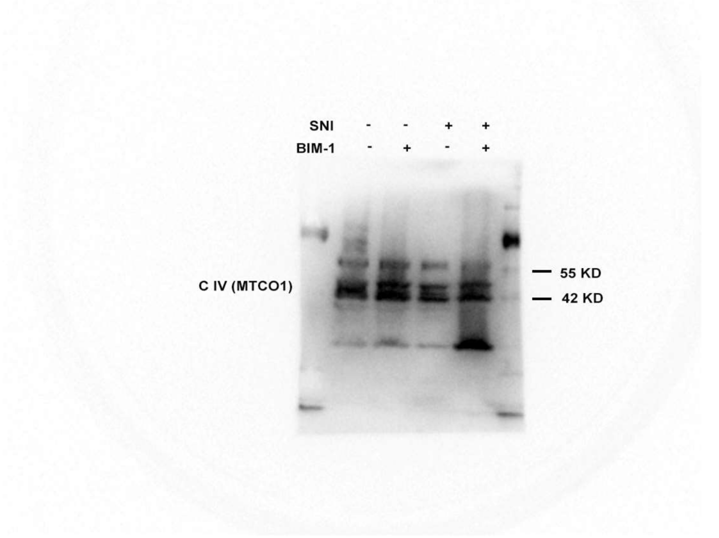

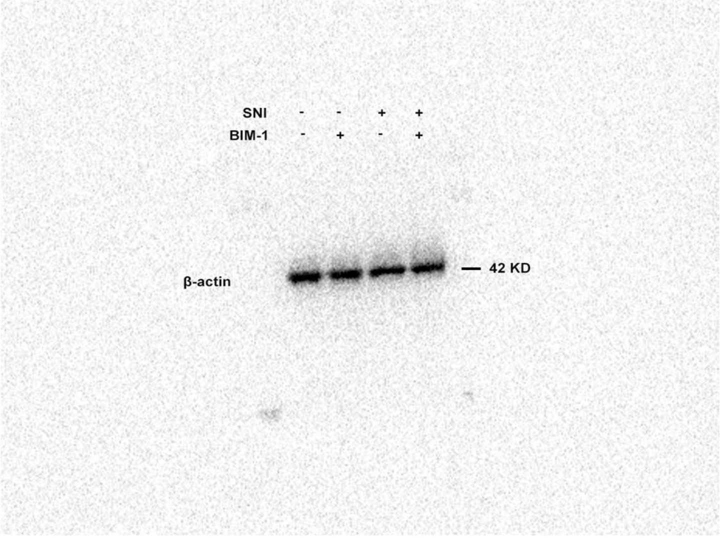

Full unedited blot for Figure 7A

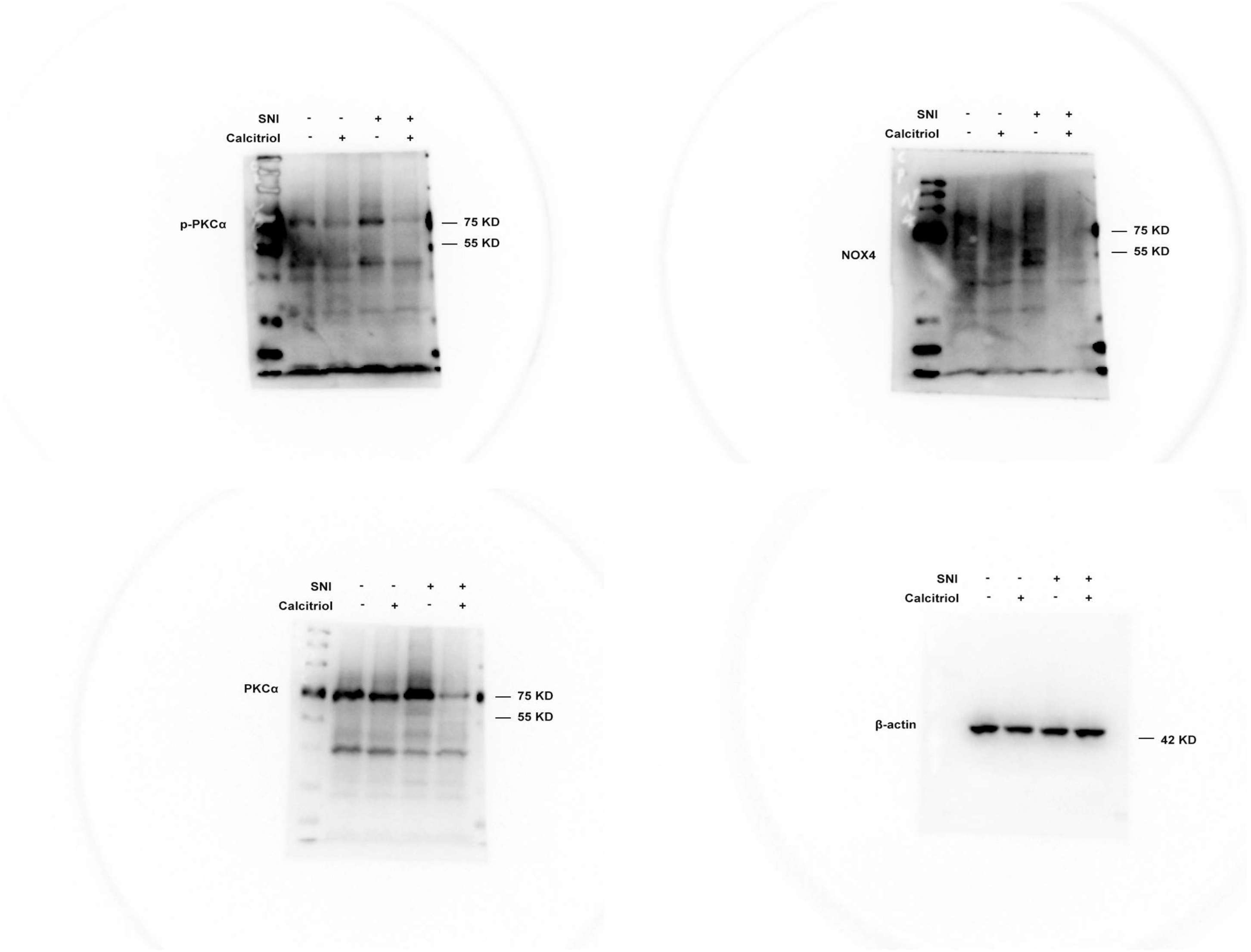

Full unedited blot for Figure 7E

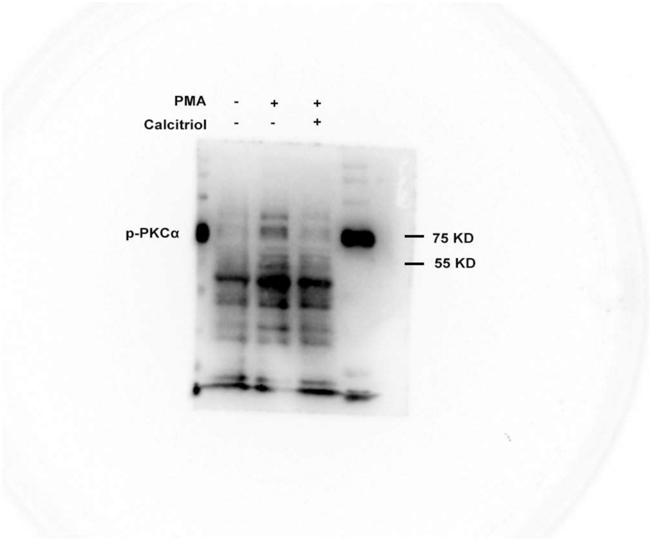

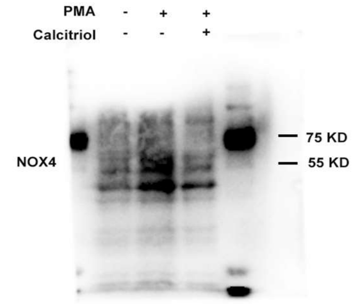

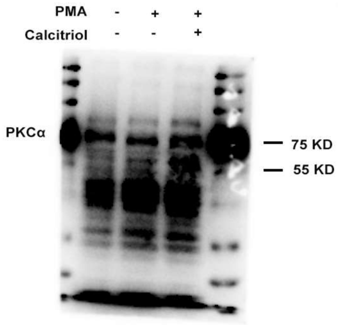

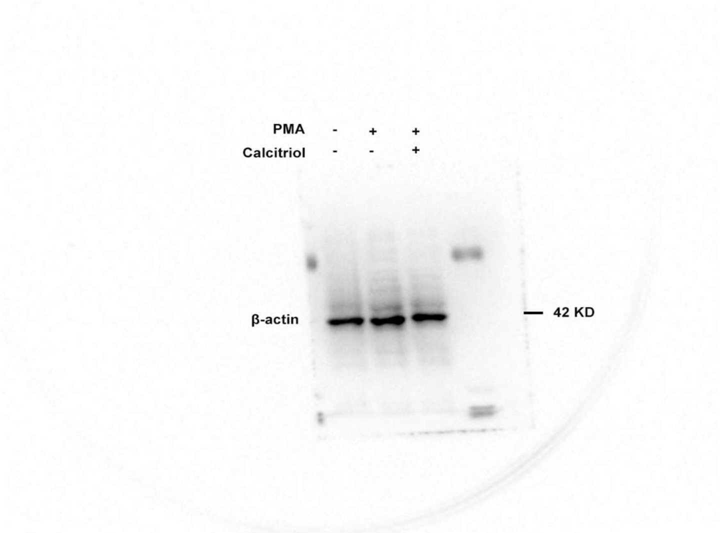

Full unedited blot for Figure 8B

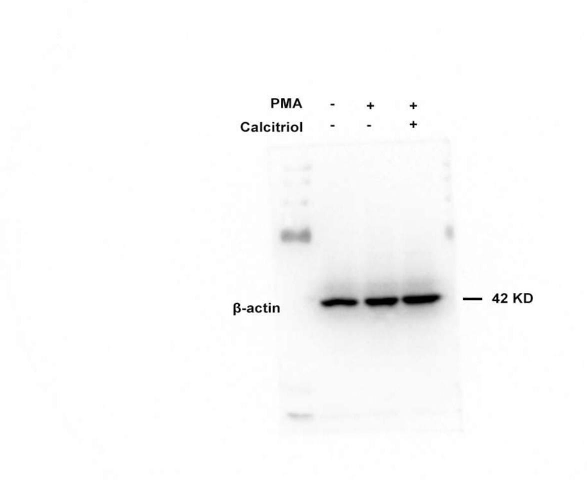

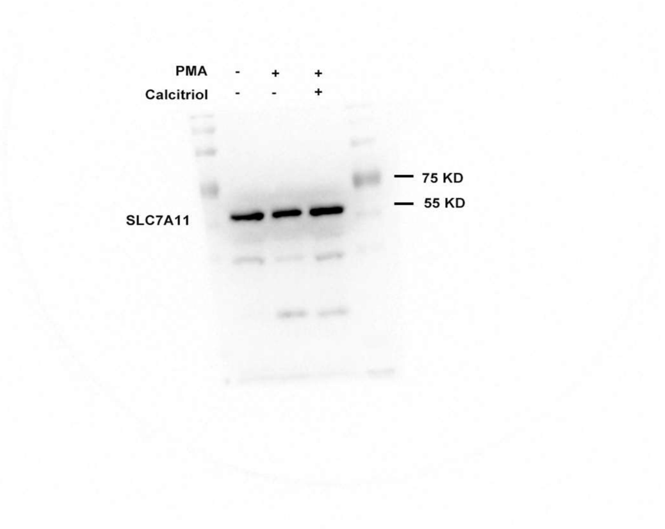

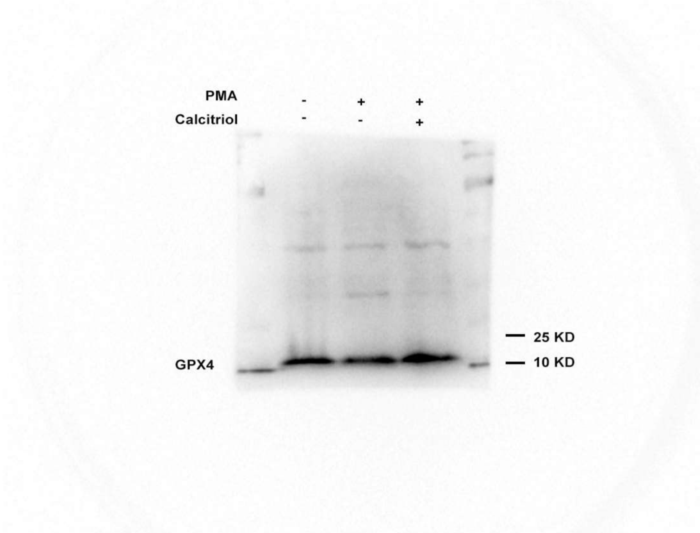

Full unedited blot for Figure 8D

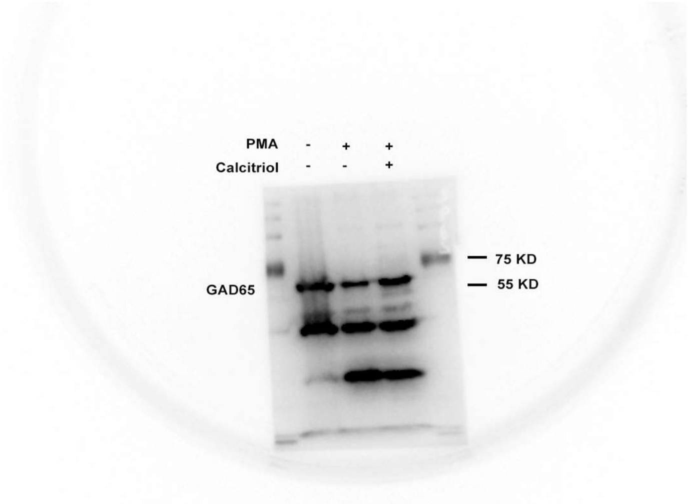

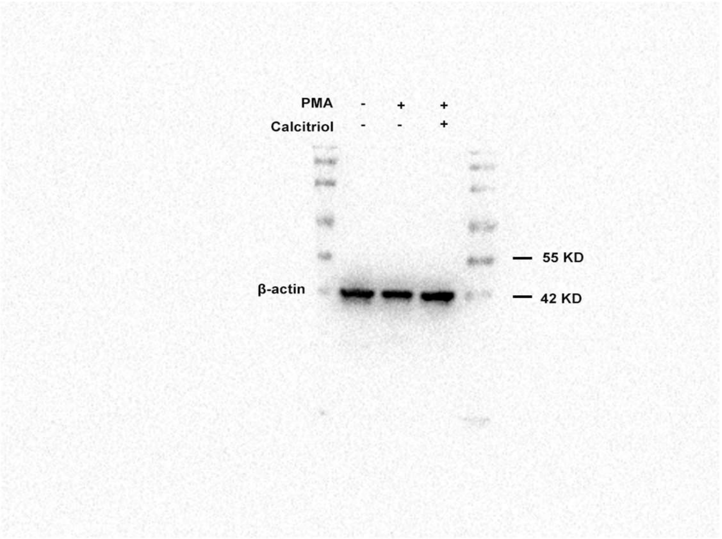

Full unedited blot for Figure 8G

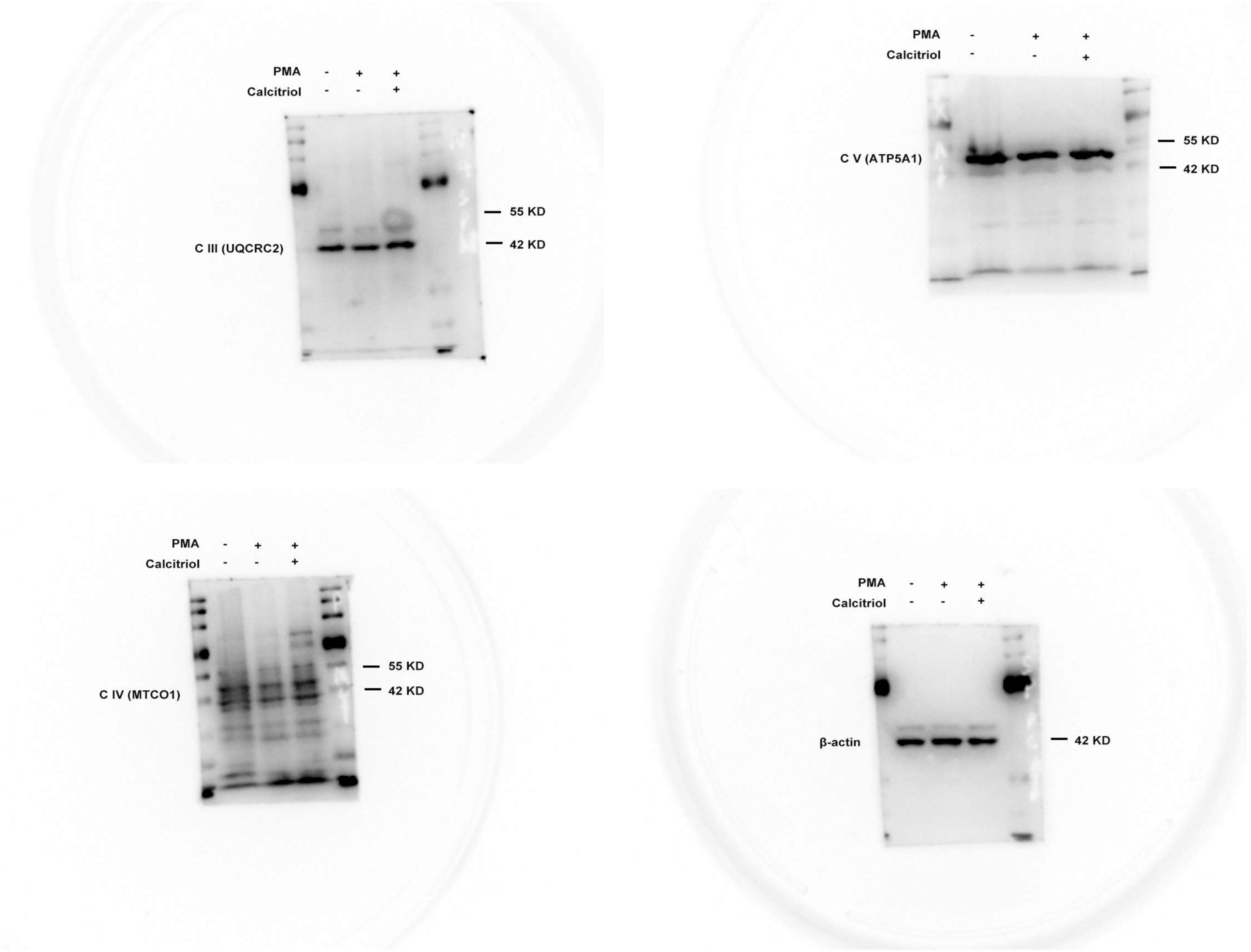

Supplement: Supplementary file 1 — Data S1. [file CNS-30-e70067-s001.pdf]
